# Reproducibility, stability, and accuracy of microbial profiles by fecal sample collection method in three distinct populations

**DOI:** 10.1371/journal.pone.0224757

**Published:** 2019-11-18

**Authors:** Doratha A. Byrd, Jun Chen, Emily Vogtmann, Autumn Hullings, Se Jin Song, Amnon Amir, Muhammad G. Kibriya, Habibul Ahsan, Yu Chen, Heidi Nelson, Rob Knight, Jianxin Shi, Nicholas Chia, Rashmi Sinha

**Affiliations:** 1 Metabolic Epidemiology Branch, Division of Cancer Epidemiology and Genetics, National Cancer Institute, National Institutes of Health, Bethesda, Maryland, United States of America; 2 Microbiome Program, Center for Individualized Medicine, Mayo Clinic, Rochester, Minnesota, United States of America; 3 Health Sciences Research, Mayo Clinic, Rochester, Minnesota, United States of America; 4 Department of Pediatrics, University of California San Diego, La Jolla, California, United States of America; 5 Department of Public Health Sciences, University of Chicago, Chicago, Illinois, United States of America; 6 New York School of Medicine, New York, New York, United States of America; 7 Department of Surgery, Mayo Clinic, Rochester, Minnesota, United States of America; 8 Department of Computer Science & Engineering, University of California San Diego, La Jolla, California, United States of America; 9 Biostatistics Branch, Division of Cancer Epidemiology and Genetics, National Cancer Institute, Bethesda, Maryland, United States of America; 10 Biomedical Engineering and Physiology, Mayo College, Rochester, Minnesota, United States of America; AC Camargo Cancer Hospital, BRAZIL

## Abstract

The gut microbiome likely plays a role in the etiology of multiple health conditions, especially those affecting the gastrointestinal tract. Little consensus exists as to the best, standard methods to collect fecal samples for future microbiome analysis. We evaluated three distinct populations (N = 132 participants) using 16S rRNA gene amplicon sequencing data to investigate the reproducibility, stability, and accuracy of microbial profiles in fecal samples collected and stored via fecal occult blood test (FOBT) or Flinders Technology Associates (FTA) cards, fecal immunochemical tests (FIT) tubes, 70% and 95% ethanol, RNA*later*, or with no solution. For each collection method, based on relative abundance of select phyla and genera, two alpha diversity metrics, and four beta diversity metrics, we calculated intraclass correlation coefficients (ICCs) to estimate reproducibility and stability, and Spearman correlation coefficients (SCCs) to estimate accuracy of the fecal microbial profile. Comparing duplicate samples, reproducibility ICCs for all collection methods were excellent (ICCs ≥75%). After 4–7 days at ambient temperature, ICCs for microbial profile stability were excellent (≥75%) for most collection methods, except those collected via no-solution and 70% ethanol. SCCs comparing each collection method to immediately-frozen no-solution samples ranged from fair to excellent for most methods; however, accuracy of genus-level relative abundances differed by collection method. Our findings, taken together with previous studies and feasibility considerations, indicated that FOBT/FTA cards, FIT tubes, 95% ethanol, and RNA*later* are excellent choices for fecal sample collection methods in future microbiome studies. Furthermore, establishing standard collection methods across studies is highly desirable.

## Introduction

The human colon is host to trillions of bacteria comprising the gut microbiota. There is strong biological plausibility for the role of the gut microbiota and its metabolites in human health, particularly for diseases of the gastrointestinal tract, such as colorectal cancer[1] and inflammatory bowel conditions,[2] and other metabolic and neurological diseases.[3, 4] Providing strong evidence for the role of the gut microbiome in human health requires fecal sample collection in large population-based, prospective epidemiologic studies; however, little consensus exists regarding the best, standard methodology for collection and storage of these samples, coordination of which is important for conducting pooled analyses of microbial data.

Currently, an array of different collection methods for fecal samples are being used in studies of the microbiome, each with advantages and disadvantages relating to feasibility for implementation and preservation of microbial profiles. While immediately freezing samples at -80°C is widely viewed as optimal,[5–9] for large epidemiologic studies this may be highly infeasible. For example, previous large microbiome studies, such as the American Gut Project,[10, 11] relied on samples collected by participants in the comfort of their home, and as a result, fecal samples spent several hours to days stored in home freezers and/or at room temperature during shipping. Ideally, the sampling method of choice should be one that preserves the microbial profile, especially under suboptimal conditions that are typical of field studies, and furthermore, that can be used for other -omics, such as transcriptomics and metabolomics studies.

Previously, small studies used 16S rRNA gene sequencing to characterize the influence of the sample collection and storage on the microbial composition of fecal samples; however, larger studies, comprising diverse study population settings, are needed to understand the best methodology for collecting and storing fecal samples in large studies of the microbiome. Herein, we meta-analyzed microbial data from four previous studies (N = 132)[12–15] spanning two countries, the United States (US) and Bangladesh, and assessed technical reproducibility, stability over 4–7 days at ambient temperature, and accuracy of the microbial profiles in fecal samples collected using six methods—no-solution, fecal immunochemical tests (FIT), fecal occult blood test (FOBT) cards or Flinders Technology Associates (FTA) cards, 70% and 95% ethanol, and RNA*later*. These analyses were based on ten microbiome metrics and the most dominant genera, providing an extensive characterization of overall microbial composition by each collection method.

## Methods

### Study population

This analysis included samples from a total of 132 healthy volunteers, drawn from four previously published studies,[12–15] that contributed fecal samples for analyses. As previously described, 20 samples were collected in the Mayo 1 study in Rochester, Minnesota, US and analyzed at both the Knight laboratory and the Mayo laboratory,[12] 52 samples were collected in the Mayo 2 study,[14] 50 samples were collected in the Bangladesh Health Effects of Arsenic Longitudinal Study (HEALS),[15] and 10 samples were collected at the University of Colorado.[13] Each participant provided written informed consent, and each study was granted ethics approval from the relevant institutional review boards as described in the original publications. The approval number from the NCI Office of Human Subjects Research for Mayo I and 2 studies is 12189 and for the Bangladesh study is 12741. The Colorado study was done under University of Colorado Boulder IRB Protocol #0409.13.

### Fecal sample collection methods

The fecal sample collection methods for each study were described in detail previously,[12–15] and the number of replicates aliquoted for each collection method and day of freezing are listed in **Table 1**. Briefly, each participant collected stool and delivered it to the study coordinator for immediate processing. The fecal specimens were mixed manually using a spatula, and aliquoted to each of the six different collection methods–no-solution, FIT tubes, FOBT/FTA cards, 70% and 95% ethanol, and RNA*later* in a random order for each participant. Approximately 1–2 grams of feces (about a full scoop) were placed in a Sarstedt feces tube (Numbrecht, Germany) containing no-solution, 2.5 mL of RNA*later* Stabilization Solution, or 2.5 mL of 70% or 95% ethanol (Sigma-Aldrich, St. Louis, Missouri). A portion of the mixed fecal specimen was also smeared thinly onto Whatman FTA cards and onto the Triple-slide Hemoccult II Elite Dispensapak Plus for FOBT (Beckman Coulter, Brea, California) per manufacturer instructions. For the FIT tube (Polymedco, Inc., Cortlandt Manor, New York) samples, the FIT probe was dipped into the fecal specimen, placed into the tube, and the tube shaken.

**Table 1:** Number of replicate samples per participant by fecal collection method and day of freezing in the meta-analyses of pooled samples from five studies (Mayo 1-Knight lab, Mayo 1-Mayo lab, Mayo 2, Bangladesh, and Colorado).

	No. of aliquots frozen on:											
	Day 0^a^						Day 4^b^					
Study, number of individuals in each study	No-solution	FIT	FOBT/FTA cards^c^	70% ethanol	95% ethanol	RNA*later*	No-solution	FIT	FOBT/FTA cards^c^	70% ethanol	95% ethanol	RNA*later*
Mayo 1^d^, n = 20 [16]	3	0	3	2	0	2	3	0	3	2	0	2
Mayo 2, n = 52 [14]	3	2	3	0	2	2	0	2	3	0	2	2
Bangladesh, n = 50 [15]	2	2	3	0	2	2	0	2	3	0	2	2
Colorado^e^, n = 10 [13]	1	0	1	1	1	1	1	0	1	1	1	1

^a^ On Day 0, a total of 326 no-solution samples, 204 FIT tubes, 376 FOBT/FTA card samples, 50 70% ethanol samples, 214 95% ethanol samples, and 254 RNA*later* samples were analyzed for each sample collection method

^b^ On Day 4, the number of samples analyzed were the same as those described in footnote ‘a’, except a total of 70 no-solution samples were analyzed

^c^ FOBT cards were a triple-slide (3-window) card on day 0 or day 4. Three windows were used per card, and each window was considered a separate aliquot.

^d^ Samples were analyzed at Knight laboratory and Mayo laboratory

^e^ Samples were frozen on Day 7

Abbreviations: FIT, fecal immunochemical test tubes; FOBT, fecal occult blood test cards; FTA, Flinders Technology Associates card

To assess reproducibility, duplicate aliquots of each specimen were created for each participant within each collection method and frozen at the same timepoint. To assess stability, duplicate aliquots of each specimen were frozen at −80°C immediately (day-0), and the remaining samples were left at ambient temperature for four days and then frozen (day-4), except in the Colorado study where samples were stored at ambient temperature for 7 days and then extracted and sequenced without further storage (day-7). For the accuracy assessment, replicates of no-solution samples were frozen immediately after collection and were considered the gold standard for comparison to immediately-frozen samples collected via other collection methods.

### DNA extraction and sequencing

The Knight lab, located initially at the University of Colorado Boulder (through 2014) and then at the University of California San Diego, performed DNA extractions and sequencing for most samples in this analysis, except for the twenty samples in Mayo 1 study that were analyzed at both the Knight lab and the Mayo Clinic. We previously found differences in microbial composition of samples from the two labs,[12] as the DNA extraction and amplification procedures differed slightly as described below. Thus, the two sets of samples were considered separately in our analyses.

#### Knight lab

Methods for DNA extraction, polymerase chain reaction (PCR) amplification, and sequencing were previously described in detail.[12, 14, 15, 17] Briefly, DNA extraction, PCR amplification of the V4 region of the 16S rRNA gene, and amplicon preparation were performed as described by Caporaso et al.[17], using the universal bacterial primer set 515F/806R,[17, 18] and can be found on the Earth Microbiome Project website (http://www.earthmicrobiome.org/emp-standard-protocols/dna-extraction-protocol/). All DNA extraction and PCR amplification included no-template controls. All barcoded amplicons were pooled in equal concentrations for sequencing on Illumina’s MiSeq for Mayo 1 (University of Colorado, Boulder, USA) and HiSeq for the Mayo 2, Bangladesh, and Colorado studies (University of California San Diego’s Institute for Genomic Medicine; 150bp). The average coverage was ~30,000–37,000 reads per sample.

#### Mayo lab

Methods for DNA extraction, PCR amplification, and sequencing were described previously.[12] Briefly, DNA was extracted using the PowerSoil DNA isolation kit (MoBio Laboratories), and the V3–V5 region (357F/926R) of the 16S rRNA gene was amplified. The samples were sequenced using the Illumina MiSeq (San Diego, CA) sequencing platform (2x250bp). The average coverage was ~70,000 reads per sample.

### Bioinformatic data processing

Reads were demultiplexed and quality filtered using QIIME 1.9 at the default setting, which was a Phred quality score of >3.[19] Each sample was independently cleaned by removing all candidate read-errors using deblur.[20] The cleaned read files were joined to make a single biom table, with each operational taxonomic unit (OTU) representing a unique 150–base pair sequence for Knight laboratory samples and 250-basepair sequence for the Mayo laboratory. All data were rarefied to 10,000 reads per sample. While the primer sets used for amplification in both the Knight and Mayo lab measure both bacteria and archaea, we focused solely on bacteria, and assigned taxonomy using the QIIME assign_taxonomy command with the rdp method and Greengenes 13.8 at 97% similarity.[13]

Next, using the R Phyloseq package,[21] we calculated two alpha diversity metrics: the number of OTUs present in a sample, which reflects species richness, and the Shannon Diversity index, which reflects both richness and evenness. We calculated four beta diversity metrics using the R vegan package[22] to reflect the shared diversity between bacterial populations in terms of ecological distance within each study population (i.e., a distance matrix was calculated for each study population): unweighted, generalized (calculated using the R GUniFrac package[23]) and weighted UniFrac distances, and the Bray-Curtis distance.

### Statistical analysis

We identified suspicious samples that were possibly contaminated or mis-labeled by calculating an outlier index based on unweighted UniFrac distance, as samples’ unweighted UniFrac values tend to cluster more tightly by subject as compared to generalized UniFrac, weighted UniFrac, and Bray-Curtis distance. Specifically, the outlier index for a given sample *K* from subject *S* was calculated as the ratio between (*a*) the average distance from sample *K* to other samples from subject *S* and (*b*) the median within-subject *S* distance. We flagged all replicate samples with an outlier index larger than 1.4 as suspicious outlier candidates. As the threshold was quite lenient some normal samples could also be flagged, so we further investigated flagged samples with principal coordinate analysis (PCoA) plots and genus-level abundance bar plots. Replicate samples that did not cluster with the rest of the samples or with a highly different genus profile were excluded.

A distance-based coefficient of determination, R^2^, was used to estimate the percentage of microbiota variability explained by subject, collection method, and storage time (‘adonis’ function in the R ‘vegan’ package[12]) using unweighted, generalized, and weighted UniFrac and Bray-Curtis distances.

To assess technical reproducibility, for each collection method, we calculated intraclass correlation coefficients (ICCs) using a mixed effects model for duplicate fecal samples frozen day-0. To assess stability at ambient temperature, for each collection method, we calculated ICCs using a mixed effects model for one randomly selected replicate of samples frozen after 4 or 7 days at ambient temperature compared to one replicate of samples frozen day-0. To assess accuracy, we calculated Spearman’s correlations (SCCs) to investigate if the rank order of microbial metrics were preserved for each randomly selected replicate for each collection method compared to the “gold standard” (i.e., fecal samples frozen day-0 with no solution). The ICCs/SCCs for stability and accuracy were averaged over 100 random samplings of replicates. For all ICC values, except distance-based ICCs (described below), we calculated 95% confidence intervals (95% CIs) using the R ‘ICC’ package (CI = ‘Smith’). For distance-based ICCs and SCCs, we calculated 95% CIs using 1,000 bootstrap samples. We interpreted ICC/SCC values <40% as poor, 40%-59.99% as fair, 60–74.99% as good, and ≥ 75% as excellent.[24]

To encompass changes in the entire microbial community, we calculated ICCs/SCCs based on ten microbial metrics: the relative abundance of the top four most abundant phyla (*Actinobacteria*, *Bacteroidetes*, *Firmicutes*, and *Proteobacteria*), two alpha diversity metrics (observed OTUs and Shannon diversity), and four beta diversity metrics (unweighted UniFrac, generalized UniFrac, weighted UniFrac, and the Bray-Curtis distance). We also calculated ICCs/SCCs for fecal genera with a prevalence in the population >80% and a mean relative abundance >0.2%. For the four beta diversity metrics, to reflect the preservation of the inter-sample relationships, we used a distance-based ICC, for which the within-subject squared distances and between-subject squared distances were used to calculate the biological and technical variance.[15] SCCs were calculated using all pairwise distances. For the relative abundance metrics, ICCs were calculated based on square root transformed relative abundance to reduce the influence of extremely high abundances and to make the data roughly meet the normality assumption under the mixed effects model for ICCs. To address the compositional nature of microbial data, we also calculated relative abundance based on the centered log-ratio transformation.[25]

Finally, we performed differential abundance analyses on the phylum, class and genus level taxa with a prevalence > 50% and a mean count > 10 reads (except for Fusobacteria, which was of biological interest and a strong risk factor for aggression and progression of colorectal cancer [26]) by comparing the abundance of each taxa for each collection method on day-4 vs. day-0 (stability), and for each collection method compared to the gold standard (accuracy). We fitted a generalized mixed effects model (GLMM, R ‘lme4’ package v1.1.14, ‘glmer’ function with ‘Poisson’ family and log link) to the taxa count data, accounting for within-subject correlation and over-dispersion.[27] To address the compositional nature, we used the log geometric mean of the counts as the sample-specific offset in the model after adding a pseudo-count of 1, which essentially assumes a linear link between the central log ratio transformed taxa abundance and the covariate. The coefficient of the GLMM is interpreted as the log-fold change of the abundance between condition.

To synthesize ICC/SCC estimates and log-fold changes across data sets, we used a meta-analytic random effects model with restricted maximum-likelihood estimation of the variance components (‘rma’ in R ‘metafor’ package). Individual estimates and their standard errors were supplied as the input.[28] The between-study variance was quantified using ‘tau2’ from ‘rma’ (estimated variance of the random effects), and the within-study variance was quantified based on the standard errors of individual ICC/SCC or log-fold change estimates.[29] Forest plots were used to visualize the results.

All statistical analyses were conducted using R (3.1.2).

## Results

Based on the outlier analyses, we excluded 0.25% (N = 1) of samples from Mayo 1- Knight laboratory, 1.25% (N = 5) of samples from Mayo 1—Mayo laboratory, 1.5% (N = 16) of samples from Mayo 2, 1.5% (N = 15) of samples from Bangladesh, and 3.6% (N = 5) of samples from Colorado.

Microbial variability was primarily explained by interindividual differences, which explained between 61% and 79% of variability in unweighted UniFrac distance, between 68% and 78% in generalized UniFrac distance, between 63% and 78% in weighted UniFrac distance, and between 77% and 86% in Bray–Curtis distance. Collection method and time at ambient temperature explained substantially lower variability (<10% and <5%, respectively; **S1 Fig**).

### Technical reproducibility

**Fig 1:**
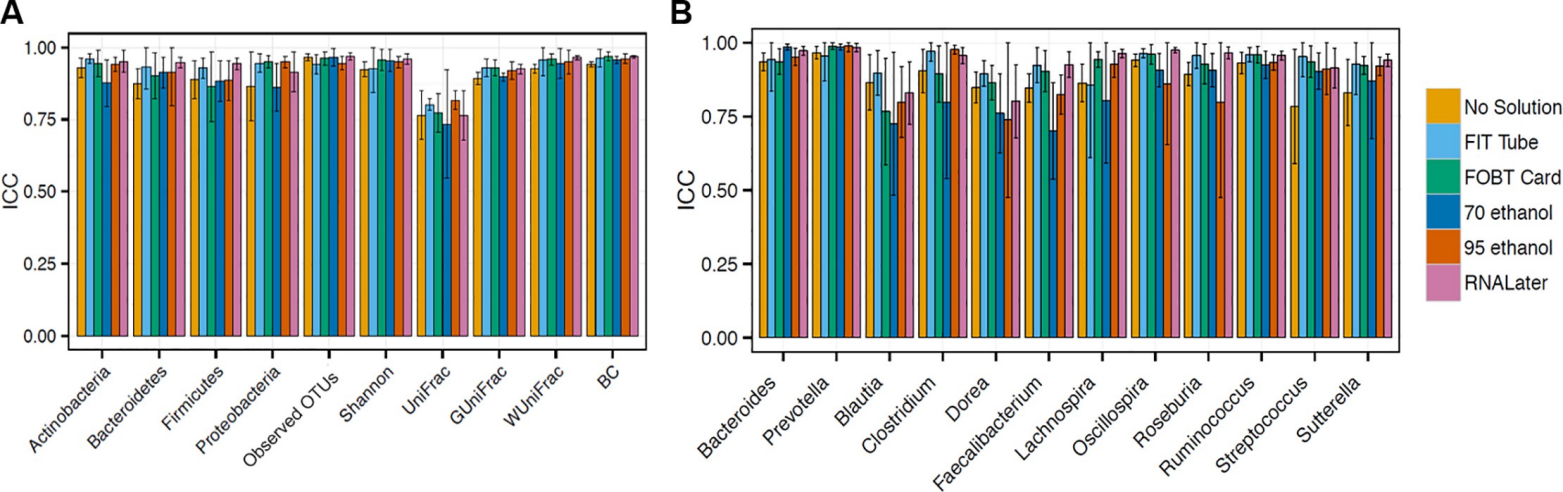
Meta-analyzed ICCs based on a random effects model for technical reproducibility comparing duplicate samples frozen day-0 from fecal samples collected using six different methods among 132 participants in five studies (Mayo 1-Knight lab, Mayo 1-Mayo lab, Mayo 2, Bangladesh, and Colorado). ICCs are based on ten microbial composition metrics (panel A; square root transformed abundance of four phyla, two alpha diversity metrics [number of observed OTUs and Shannon index] and four beta diversity metrics [unweighted UniFrac, generalized UniFrac, weighted UniFrac, and Bray-Curtis distance]), and square root transformed select bacterial genera (panel B) with prevalence in the population >80% and a mean relative abundance >0.2%. Error bars represent 95% CIs. *Abbreviations*: *BC*, *Bray-Curtis distance; FIT*, *fecal immunochemical test tubes; FOBT*, *fecal occult blood test cards; FTA*, *Flinders Technology Associates cards; GUniFrac*, *generalized UniFrac distance; ICC*, *intraclass correlation coefficient; UniFrac*, *unweighted UniFrac distance; WUnifrac*, *weighted UniFrac distance*.

The meta-analyzed ICCs for technical reproducibility comparing duplicate aliquots for each collection method frozen at day-0 for ten microbial composition metrics are shown in **Fig 1A** (see **Table A in S1 File** for exact ICCs and 95% CIs). For each collection method, reproducibility was excellent for virtually all metrics (all ICCs > 75%), and the variance within and between studies was generally minimal (**S2 Fig**). The ICCs for technical reproducibility of select genera are shown in **Fig 1B**. There were no major differences between the collection methods in reproducibility ICCs, and all were excellent–except for *Blautia* and *Faecalibacterium* (both from the Firmicutes phylum) in 70% ethanol samples and *Dorea* in 95% ethanol samples. As shown in **S3 Fig,** the reproducibility ICCs for the centered-log ratio transformed phyla and genera were similarly excellent.

### Stability

**Fig 2:**
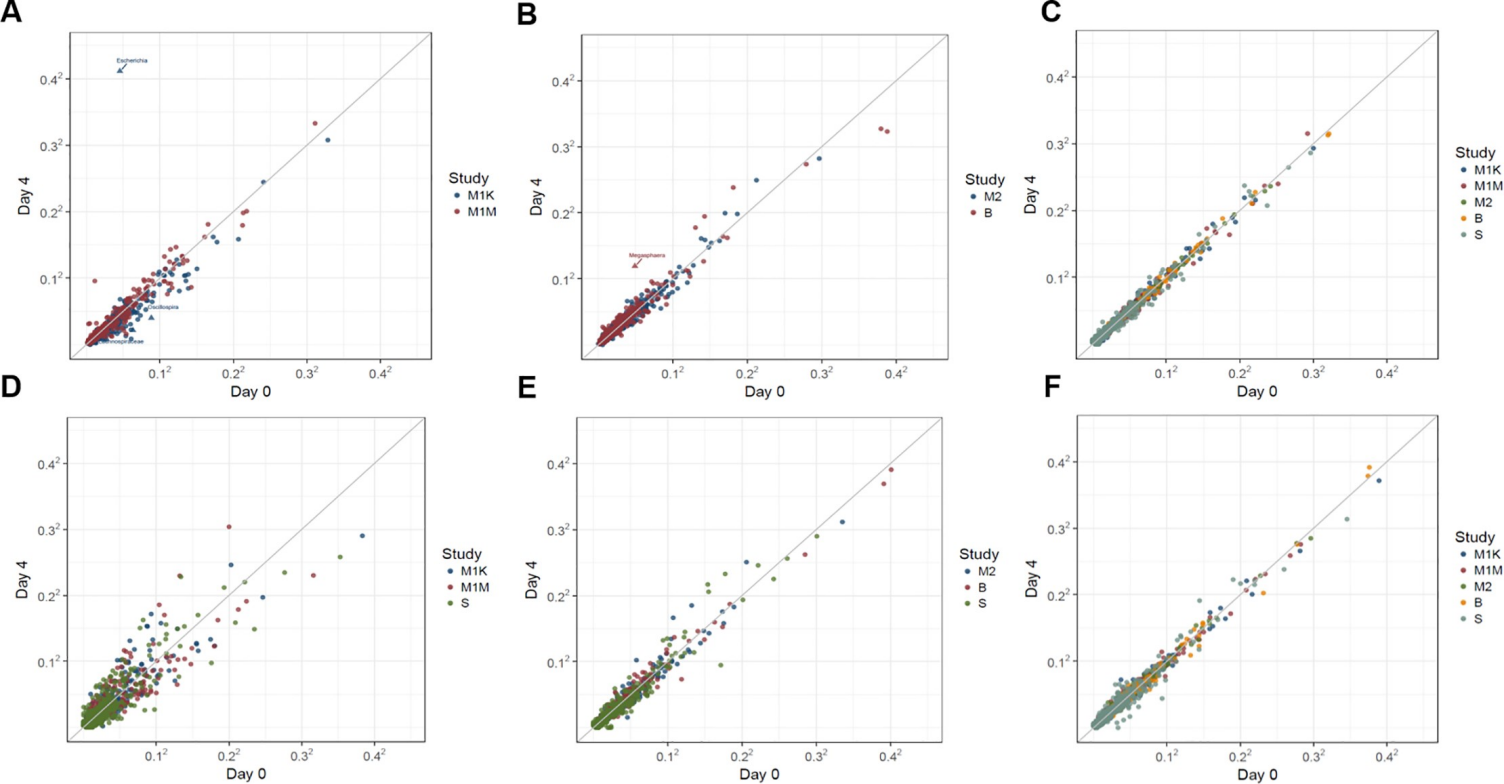
**Comparison of square root transformed mean OTU abundance between day-0 and day-4/7 of freezing for fecal samples from 132 participants in five studies (Mayo 1-Knight lab, Mayo 1-Mayo lab, Mayo 2, Bangladesh, and Colorado), collected using six different methods (A) no-solution, (B) FIT tubes, (C) FOBT cards, (D) 70% ethanol, (E) 95% ethanol, and (F) RNA*later*.** *Abbreviations*: *B*, *Bangladesh; FIT*, *fecal immunochemical test tubes; FOBT*, *fecal occult blood test cards; FTA*, *Flinders Technology Associates cards; M1K*, *Mayo 1*, *Knight Laboratory; M1M*, *Mayo 1*, *Mayo Laboratory; M2*, *Mayo 2; OTU*, *operational taxonomic unit; S*, *Colorado Study*.

**Fig 3:**
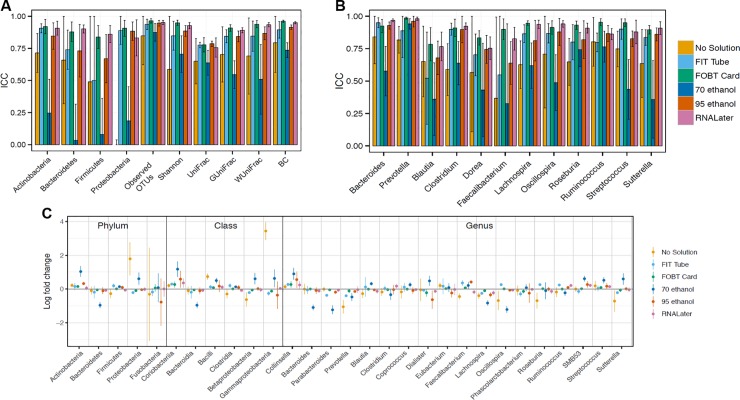
Meta-analyzed ICCs and log-fold changes based on random effects models for microbiome stability comparing fecal samples frozen on day-4/7 to those frozen at day-0 for six fecal sample collection methods among 132 participants in five studies (Mayo 1-Knight lab, Mayo 1-Mayo lab, Mayo 2, Bangladesh, and Colorado). ICCs are based on ten microbial composition metrics (panel A; square root transformed abundance of four phyla, two alpha diversity metrics [number of observed OTUs and Shannon index] and four beta diversity metrics [unweighted UniFrac, generalized UniFrac, weighted UniFrac, and Bray-Curtis distance]), and select square root transformed bacterial genera (panel B) with prevalence in the population >80% and a mean relative abundance >0.2%. Log-fold changes in relative abundance from day-0 (panel C) are based on select taxa with prevalence > 50% and a mean read count > 10. All error bars represent 95% CIs. *Abbreviations*: *BC*, *Bray-Curtis distance; FIT*, *fecal immunochemical tests tubes; FOBT*, *fecal occult blood test cards; FTA*, *Flinders Technology Associates cards; GUniFrac*, *generalized UniFrac distance; ICC*, *intraclass correlation coefficient; UniFrac*, *unweighted UniFrac distance; WUnifrac*, *weighted UniFrac distance*.

Comparisons of mean OTU abundance between samples frozen on day-0 and on day-4 (Mayo 1, Mayo 2, and Bangladesh studies) or day-7 (Colorado study) are shown for each collection method in **Fig 2**. For all collection methods, most mean OTU abundances were similar between day-0 and day-4/7 except mean abundance was less strongly correlated between days of freezing in 70% ethanol, and no-solution samples had approximately 60-fold times more abundant *Proteobacteria Escherichia* on day-4/7 compared to day-0. As shown by the stability ICCs in **Fig 3A** (see Table B in **S1 File** for exact ICCs and 95% CIs and **S4 Fig** for forest plot of ICCs) stability was excellent for all microbiome metrics in samples collected via FOBT/FTA cards and RNA*later* (ICCs ≥ 75%). Samples co1lected via FIT tubes and 95% ethanol generally had excellent stability for alpha diversity and most beta diversity measures; however, stability of phyla and select genera ranged more widely from fair to excellent (ICCs ranging from 0.50–0.91 for FIT tubes and 0.67–0.89 for 95% ethanol). For no-solution and 70% ethanol samples, the ICCs for all metrics were generally lower than the other collection methods, with fair to good stability for the alpha/beta diversity metrics, but poor stability (all ICCs <0.25) for relative abundance of the four most abundant phyla in 70% ethanol samples and for the Proteobacteria phylum in no-solution samples (ICC = 0). There were similar patterns in stability across collection methods for select genera (**Fig 3B**; e.g., good to excellent ICCs for FOBT/FTA cards, FIT tubes, 95% ethanol, and RNA*later*, and generally poor to fair ICCs for 70% ethanol samples and no-solution samples). As shown in **S5 Fig,** the ICCs for stability of the centered-log ratio transformed top four phyla and select genera were similar to their square root transformed counterparts. For example, ICCs for no-solution samples and 70% ethanol samples were similarly poor to fair for centered-log ratio transformed abundances.

The log-fold change of selected phyla, classes, and genera prevalent in at least 50% of the population with a mean count of > 10 reads (except for Fusobacteria, which was of biological interest) from day-0 to day-4/7 is shown in **Fig 3C** (see Table C in **S1 File** for exact log-fold changes, standard errors, and p-values). For 70% ethanol samples frozen on day-4/7 compared to 70% ethanol samples frozen day-0, 62% percent of taxa were statistically significantly higher or lower in abundance; whereas, for FOBT/FTA cards frozen on day-4/7 vs. frozen day-0, only 7% percent of taxa were statistically significantly higher or lower. For no-solution samples frozen on day-4/7 vs. frozen day-0, there was more than 30-fold higher Gammaproteobacteria abundance (*p =* 6.02 x E^-38^). There were no statistically significant differences in Fusobacteria abundance after time spent at ambient temperature for any of the collection methods.

### Accuracy

**Fig 4:**
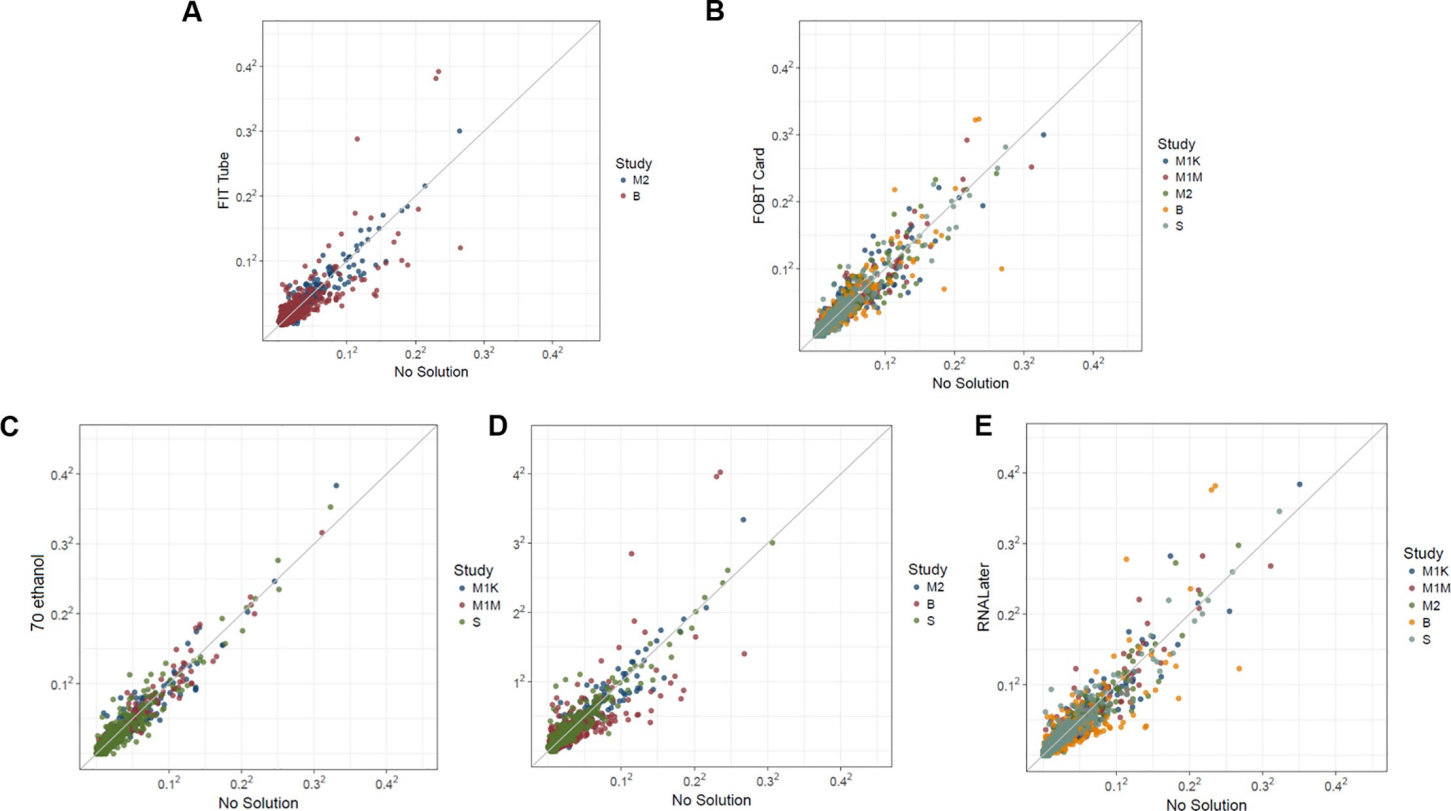
**Comparison of mean OTU abundance for each fecal sample collection method frozen on day-0 compared to no-solution samples frozen on day-0 (the gold standard) for fecal samples from 132 participants in five studies (Mayo 1-Knight lab, Mayo 1-Mayo lab, Mayo 2, Bangladesh, and Colorado), collected using five different methods (A) FIT tubes, (B) FOBT cards, (C) 70% ethanol, (D) 95% ethanol, and (E) RNA*later*.** *Abbreviations*: *B*, *Bangladesh; FIT*, *fecal immunochemical tests; FOBT*, *fecal occult blood test cards; FTA*, *Flinders Technology Associates cards; M1K*, *Mayo 1 Knight Laboratory; M1M*, *Mayo 1*, *Mayo Laboratory; M2*, *Mayo 2; OTU*, *operational taxonomic unit; S*, *Colorado Study*.

**Fig 5:**
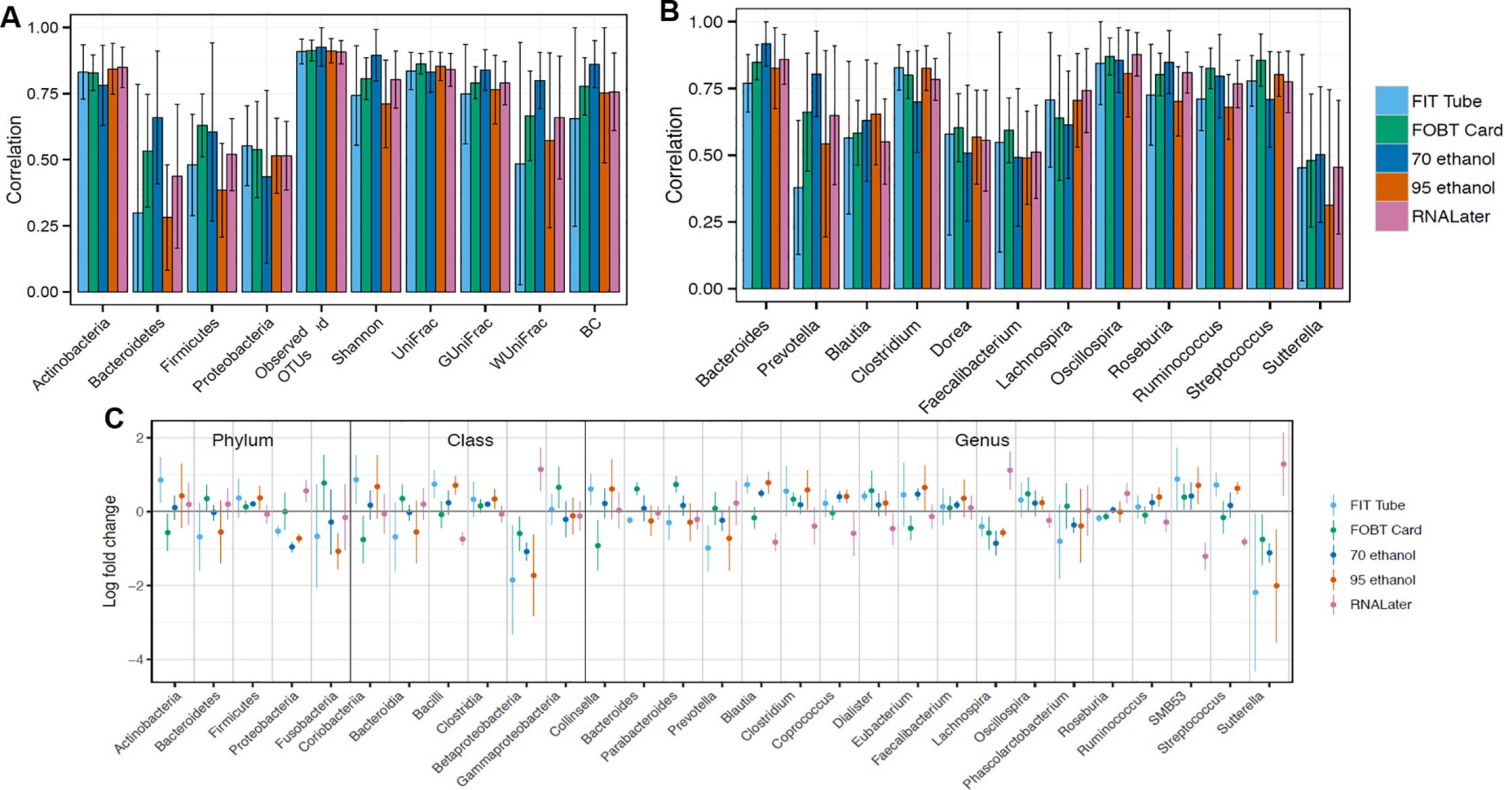
Meta-analyzed SCCs and log-fold changes based on random effects model for accuracy of each collection method compared to no-solution samples frozen on day-0 (the gold standard) among 132 participants in five studies (Mayo 1-Knight lab, Mayo 1-Mayo lab, Mayo 2, Bangladesh, and Colorado) with fecal samples collected using six different methods. SCCs are based on ten microbial composition metrics (panel A; square root transformed abundance of four phyla, two alpha diversity metrics [number of observed OTUs and Shannon index] and four beta diversity metrics [unweighted UniFrac, generalized UniFrac, weighted UniFrac, and Bray-Curtis distance]), and square root transformed select bacterial genera (panel B) with prevalence in the population >80% and a mean relative abundance >0.2%. Log-fold changes in relative abundance from day-0 (panel C) are based on select taxa with prevalence > 50% and a mean read count > 10. All error bars represent 95% CIs. *Abbreviations*: *BC*, *Bray-Curtis distance; FIT*, *fecal immunochemical test tubes; FOBT*, *fecal occult blood test cards; FTA*, *Flinders Technology Associates cards; GUniFrac*, *generalized UniFrac distance; SCC*, *Spearman correlation coefficient; UniFrac*, *unweighted UniFrac distance; WUnifrac distance*, *weighted UniFrac distance*.

To evaluate the accuracy of the fecal sample collection methods, we compared microbiome metrics in samples frozen on day-0 for each collection method to no-solution samples frozen on day-0 (the putative gold standard). As shown in **Fig 4**, the mean abundance of OTUs was consistently relatively concordant with the gold standard for all collection methods. We found that for most collection methods, SCCs for concordance with the gold standard were generally good to excellent for alpha and beta diversity estimates (**Fig 5A** and **5B**; see Table D in **S1 File** for exact SCCs and 95% CIs and **S6 Fig** for forest plot of SCCs**)**, but that weighted UniFrac SCCs were generally lower (fair to good SCCs). Furthermore, for most collection methods, the relative abundances of dominant phyla were generally poorly to fairly concordant with the gold standard, except for Actinobacteria, which was excellently concordant for all collection methods. As shown in **S7 Fig,** the SCCs for accuracy of the centered-log ratio transformed top four phyla and select genera were similar to those of their square root transformed counterparts.

The log-fold change of selected phyla, classes, and genera prevalent in at least 50% of the population with a mean count of > 10 reads for each collection method compared to samples with no-solution frozen day-0 (the gold standard) is shown in **Fig 5C**. Compared to no-solution samples frozen day-0, 31%, 28%, 24%, 24%, and 10% of taxa in 70% ethanol, RNA*later*, FIT tubes, 95% ethanol, and FOBT/FTA card samples were statistically significantly more or less abundant, respectively (see Table E in **S1 File** for exact log-fold changes, standard error, and p-values). The only collection method with a statistically significantly lower abundance in Fusobacteria than the gold standard was 95% ethanol, which had 66% lower Fusobacteria abundance.

**Fig 6:**
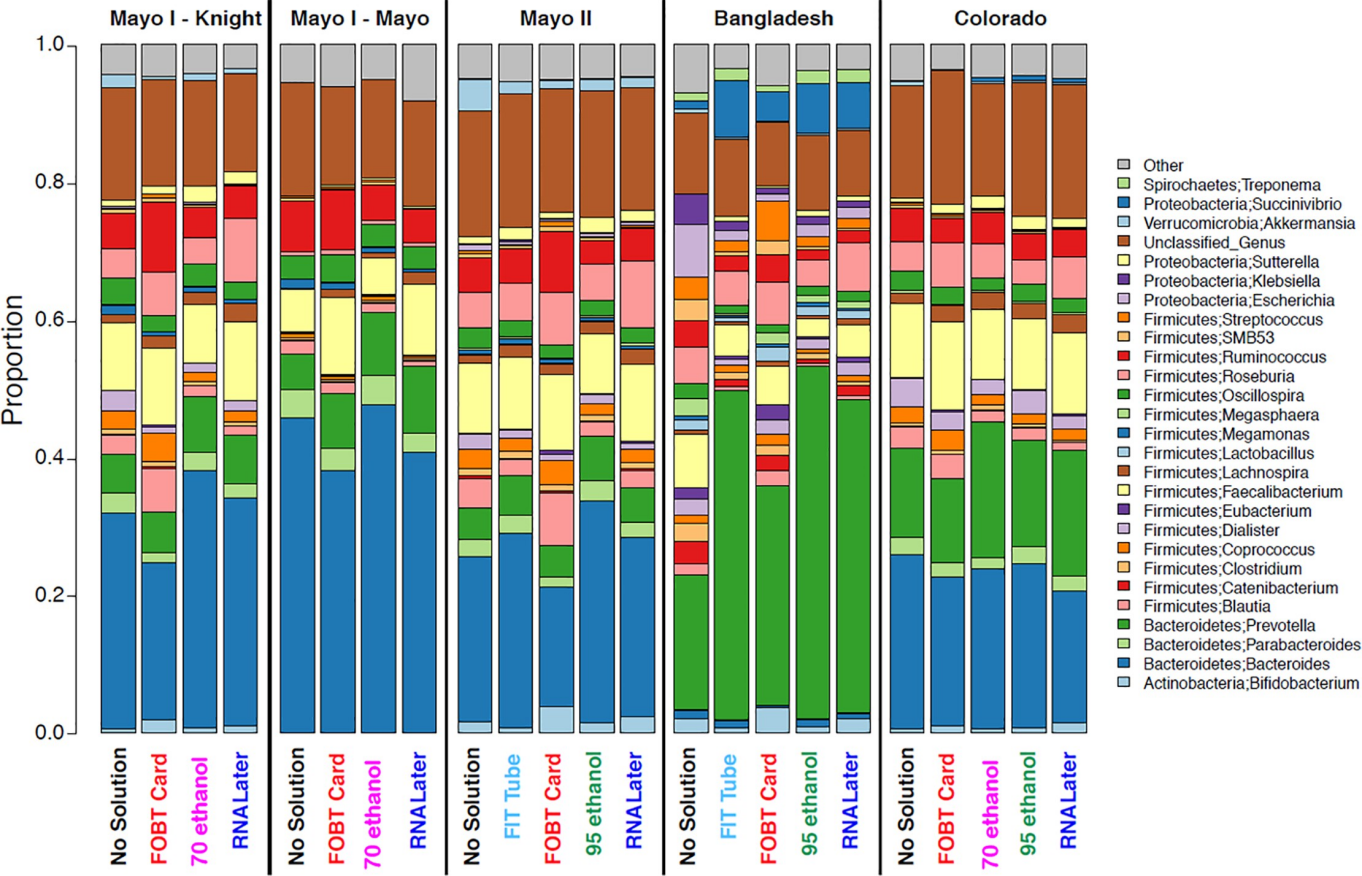
Relative abundance of bacterial genera in fecal samples frozen on day-0, collected from 132 participants in five studies (Mayo 1-Knight lab, Mayo 1-Mayo lab, Mayo 2, Bangladesh, and Colorado). OTU counts were merged across samples by study population and collection method, and relative abundance was calculated for the merged samples. OTUs that could not be assigned to a specific genus were combined into “Unclassified_Genus”. The ‘Other’ group comprised all genera with mean abundance less than 0.5%. *Abbreviations*: *FIT*, *fecal immunochemical test tubes; FOBT*, *fecal occult blood test cards; FTA*, *Flinders Technology Associates cards; OTU*, *operational taxonomic unit*.

The relative abundance of genera detected in the fecal samples collected from 132 study participants in the three distinct populations (Mayo, Bangladesh, and Colorado) by collection method on day-0 of freezing is presented in **Fig 6.** There were marked differences in the distribution of genera between the Bangladesh and US study populations–for example, fecal samples in the Bangladesh study were more abundant in *Prevotella* (average relative abundance = 38.6%) compared to US populations (average relative abundance = 0.09%). Clearly, within each study population, the general distribution of abundance of each genus was relatively inconsistent across sample collection method.

## Discussion

In the largest summary study of bacterial profile reproducibility, stability, and accuracy of six fecal collection methods to date, our results support that, for ten microbiome metrics and select genera, which taken together comprehensively characterize the microbial profile and are measures routinely used in microbiome-exposure/disease analyses: 1) all six of the fecal sample collection methods had excellent technical reproducibility; 2) all collection methods, except for no solution and 70% ethanol, had good to excellent stability; and, 3) compared to the no-solution, immediately-frozen samples, all collection methods generally had fair to excellent accuracy. Below we discuss these findings in context with their implications for the collection of fecal samples to study the microbiome in large-scale studies, and the need for standardization of collection methods across studies to facilitate pooling of microbial data.

We conducted an extensive literature search and summarized the rationale for use, feasibility considerations, and previous findings related to microbial reproducibility, stability, and accuracy for each collection method in **S1 Table**. Each collection method has advantages and disadvantages pertaining to the stabilization of DNA, prevention of bacterial growth, and preservation of a microbial profile comparable to the immediately-frozen, no-solution gold standard. As demonstrated by our findings and previous findings, no solution and 70% ethanol are less stable collection methods compared to others when stored at ambient temperature, likely explained by the total lack of (or dilution of in 70% ethanol) DNA-stabilizing and anti-microbial properties. For example, while some studies found microbiome metrics were stable in samples stored in no solution up to three days at ambient temperature,[30, 31] others found alterations in relative abundances of major taxa,[5, 9, 32] lower alpha diversity and lower bacterial counts after 8–24 hours[33, 34] or three days,[5] and greater weighted UniFrac, unweighted UniFrac, and Bray-Curtis distances from immediately-frozen samples.[32, 35] Furthermore, we observed a drastic growth of Gammaproteobacteria in no-solution samples after 4/7 days at ambient temperature, which may be concerning in gut health analyses because of the association of Gammaproteobacteria with inflammatory bowel disease.[36] However, as described previously, these taxa tend to grow well at room-temperature and may result from contaminated storage conditions, but importantly can be filtered using Deblur.[36] Fecal samples stored in FIT tubes were previously found to be moderately to excellently well-preserved at ambient temperature and comparable to immediately-frozen, no-solution samples.[37, 38] However, some studies found differences in relative abundances of phyla and genera,[37] lower Shannon diversity with longer storage time at ambient temperature,[38] and compared to the gold standard, lower numbers of observed OTUs and differences in overall composition based on Bray-Curtis distance.[39] Multiple studies found that microbial profiles of fecal samples from FOBT/FTA cards were moderately to excellently stable at ambient temperature over the course of multiple days[7, 39–42] and comparable to the gold standard;[43] although, one study found a lower DNA yield among fecal samples from monkeys collected on FTA cards after 8 weeks.[44] Fewer studies investigated the stability/accuracy of human fecal samples stored in 70% and 95% ethanol. A study of tissue samples stored in 95% ethanol and another of gorilla fecal samples stored in 96% ethanol both found a lower DNA yield than fresh samples;[45, 46] whereas, another study of monkey fecal samples found that 100% ethanol preserved bacterial composition and diversity well compared to fresh fecal samples.[38, 44] Previous findings were mixed for RNA*later* stability and accuracy.[5, 7, 13, 40, 43, 45, 47–50] For example, Flores et al.[51] found that microbial profiles of fecal samples collected in RNA*later* were generally stable up to 7 days at ambient temperature prior to freezing; however, other studies found relatively large bacterial community shifts after just 3 days at ambient temperature[50] or lower alpha diversity and DNA purity after 3 days to a week at ambient temperature.[5, 7, 49]

Given the gut microbiome’s highly plausible role in health–particularly gut health–powerful epidemiologic studies of the microbial profile in relation to health outcomes will likely require that microbial data be pooled from multiple studies to observe differences, particularly at the genus level. The variability we observed in the genus-level relative abundances across collection methods indicates that it is optimal for researchers to coordinate the use of at least one collection method commonly used in other studies; however, we also observed that inter-individual differences explained a much higher percentage of microbial variability than collection method. Pooling microbial data from fecal samples collected via different methods could theoretically be acceptable with careful methodological consideration (e.g., when appropriate, ensuring case and control samples are collected via same collection methods, adjusting for collection method in multivariable regression models, etc.); however, the power to detect and interpretation of microbiome-disease associations may be affected since effect sizes of microbiome-disease and microbiome-collection method associations may be similarly moderate. For example, Shah et al. meta-analyzed microbial data from multiple studies to identify microbial markers associated with colorectal cancer and found that samples clustered primarily by their original studies rather than colorectal cancer case/control status due to differences between studies in sample collection and DNA extraction methods/16s rRNA sequencing region, reducing their ability to detect certain microbiome-colorectal cancer associations.[52]

When conceptualizing the implications of findings from this study, it is important to not only consider the collective findings described above, but also place them in context with cost/feasibility for implementation in large-scale studies and use for other -omics (outlined in **S1 Table**). For example, in this study, the fecal samples in RNA*later* were collected in 2.5 mL of solution, which some researchers previously indicated may not be adequate volume for microbial stability and accuracy;[53] however, microbial profiles of RNA*later* samples were generally excellently stable and accurate, and compared to larger volumes, 2.5 mL is likely more feasible for both cost and storage requirements in large cohort studies.[54] But better yet, FOBT/FTA cards are widely used for colorectal cancer screening, are easily transportable and storable, and are less than half of the cost of RNA*later*. In terms of use for other -omics, RNA*later* cannot be used for metabolomics,[55] but can be used metagenomic and metatranscriptomic studies as it was previously found to maintain similar metagenomic and metatranscriptomic profiles to gold standard samples, along with 95% ethanol samples.[12, 47, 49] Both 95% ethanol and FOBT/FTA cards were found to be reproducible, stable, and accurate in metabolomics studies[43, 55]–more so than FIT tubes.[55]

This study has several limitations. First, the microbial metrics discussed herein were based on characterizations of 16S rRNA gene sequences of bacteria and use of shotgun metagenomics is becoming more widespread; however, as of now, 16S rRNA sequencing is an affordable, widely-used method to characterize the bacterial microbiome, and studies using other assays may find these results helpful in selecting fecal collection methods. Second, Mayo 1 and Knight laboratories used different DNA extraction and amplification protocols, which produced variability in microbial metrics; however, we conducted stratified analyses by lab and found no meaningful differences in conclusions for each sample collection method. Third, overall there was large variance between studies, especially for phylum- and genus-level analyses; however, the conclusion for each sampling method remained consistent across study populations.

This analysis also has several important strengths. First, with 132 participants from diverse populations, there was greater statistical power to detect differences between collection methods than any previous study comparing fecal collection methods. Second, this analysis included a wide variety of collection methods ranging in cost and ease of implementation, and included FIT, which is gaining popularity for CRC screening. Third, our study population was heterogeneous and included samples gathered in a low-to-middle income country, Bangladesh, where relevant conditions may differ from those present in the US; yet and still, the results in this study were similar to those of the US populations.

## Conclusions

These findings, taken together with previous literature and feasibility considerations, indicate that FOBT/FTA cards, FIT tubes, RNA*later*, *and* 95% ethanol samples may be an appropriate choice to collect fecal samples for the measurement of microbiome data in future studies, as these options are reproducible, stable, and relatively accurate. As the gut microbiome becomes increasingly recognized for its role in the etiology of gut and systemic health, it is imperative to characterize microbial profiles using the best available methodologies, and to move toward standardization of fecal sample collection across study populations. Future studies should further investigate the long-term stability (over the course of years) of these collection methods, similar to those samples stored in biobanks, and continue to explore the stability, accuracy, and reproducibility of each fecal collection method for other -omics, such as shotgun metagenomics.

## Supporting information

S1 Fig Averaged distance-based coefficient of determination R^2^ (unweighted UniFrac, generalized UniFrac, weighted UniFrac, and Bray-Curtis distance) quantifying the percentage of microbiota variability based on subject, collection method, or day of freezing. Error bars represent the standard deviation across all the studies. *Abbreviations*: *UniFrac*, *unweighted UniFrac; gUniFrac*, *generalized UniFrac; wUniFrac*, *weighted UniFrac; BC*, *Bray-Curtis distance*.
(TIF)

S2 Fig Forest plot showing reproducibility ICCs for sample collection methods, stratified by study, for each of ten different microbial composition metrics [abundance of four phyla, two alpha diversity metrics (number of observed OTUs and Shannon index) and four beta diversity metrics (unweighted UniFrac, generalized UniFrac, weighted UniFrac, and Bray-Curtis distance)]. *Abbreviations: B, Bangladesh; FIT, fecal immunochemical tests; FOBT, fecal occult blood test cards; FTA. Flinders Technology Associates (FTA) cards; M2, Mayo Clinic 2 study; M1M, Mayo Clinic 1 study, Mayo Clinic lab; M1K, Mayo Clinic 1 study, Knight lab; META, meta-analysis (pooled samples)*.
(TIF)

S3 Fig Meta-analyzed ICCs based on a random effects model for technical reproducibility comparing duplicate samples frozen day-0 from fecal samples collected using six different methods among 132 participants in five studies (Mayo 1-Knight lab, Mayo 1-Mayo lab, Mayo 2, Bangladesh, and Colorado). ICCs are based on ten microbial composition metrics (panel A; centered log-ratio transformed abundance of four phyla, two alpha diversity metrics [number of observed OTUs and Shannon index] and four beta diversity metrics [unweighted UniFrac, generalized UniFrac, weighted UniFrac, and Bray-Curtis distance]), and centered log-ratio transformed select bacterial genera (panel B) with prevalence in the population >80% and a mean relative abundance >0.2%. Error bars represent 95% CIs. *Abbreviations*: *BC*, *Bray-Curtis distance; FIT*, *fecal immunochemical test tubes; FOBT*, *fecal occult blood test cards; FTA*, *Flinders Technology Associates cards; GUniFrac*, *generalized UniFrac distance; ICC*, *intraclass correlation coefficient; UniFrac*, *unweighted UniFrac distance; WUnifrac*, *weighted UniFrac distance*.
(TIF)

S4 Fig Forest plot showing stability ICCs (stability over 4 or 7 days at ambient temperature) for sample collection methods, stratified by study analyzed for ten microbial composition metrics [abundance of four phyla, two alpha diversity metrics (number of observed OTUs and Shannon index) and four beta diversity metrics (unweighted UniFrac, generalized UniFrac, weighted UniFrac, and Bray-Curtis distance)]. *Abbreviations: B, Bangladesh; FIT, fecal immunochemical tests; FOBT, fecal occult blood test cards; FTA. Flinders Technology Associates (FTA) cards; M2, Mayo Clinic 2 study; M1M, Mayo Clinic 1 study, Mayo Clinic lab; M1K, Mayo Clinic 1 study, Knight lab; S, Colorado study; META, meta-analysis (pooled samples)*.
(TIF)

S5 Fig Meta-analyzed ICCs based on random effects models for microbiome stability comparing fecal samples frozen on day-4/7 to those frozen at day-0 for six fecal sample collection methods among 132 participants in five studies (Mayo 1-Knight lab, Mayo 1-Mayo lab, Mayo 2, Bangladesh, and Colorado). ICCs are based on ten microbial composition metrics (panel A; centered log-ratio transformed abundance of four phyla, two alpha diversity metrics [number of observed OTUs and Shannon index] and four beta diversity metrics [unweighted UniFrac, generalized UniFrac, weighted UniFrac, and Bray-Curtis distance]), and centered log-ratio transformed select bacterial genera (panel B) with prevalence in the population >80% and a mean relative abundance >0.2%. All error bars represent 95% CIs. *Abbreviations*: *BC*, *Bray-Curtis distance; FIT*, *fecal immunochemical tests tubes; FOBT*, *fecal occult blood test cards; FTA*, *Flinders Technology Associates cards; GUniFrac*, *generalized UniFrac distance; ICC*, *intraclass correlation coefficient; UniFrac*, *unweighted UniFrac distance; WUnifrac*, *weighted UniFrac distance*.
(TIF)

S6 Fig Forest plot showing accuracy (as assessed by Spearman’s correlation coefficients) for sample collection methods, stratified by study, for each of ten different microbial composition metrics [abundance of four phyla, two alpha diversity metrics (number of observed OTUs and Shannon index) and four beta diversity metrics (unweighted UniFrac, generalized UniFrac, weighted UniFrac, and Bray-Curtis distance)]. Samples at T = 0 were compared with flash-frozen samples. *Abbreviations*: *B*, *Bangladesh study; FIT*, *fecal immunochemical tests; FOBT*, *fecal occult blood test cards; FTA*. *Flinders Technology Associates (FTA) cards; M2*, *Mayo Clinic 2 study; M1M*, *Mayo Clinic 1 study*, *Mayo Clinic lab; M1K*, *Mayo Clinic 1 study*, *Knight lab; S*, *Colorado study; META*, *meta-analysis (pooled samples)*.
(TIF)

S7 Fig Meta-analyzed SCCs based on random effects model for accuracy of each collection method compared to no-solution samples frozen on day-0 (the gold standard) among 132 participants in five studies (Mayo 1-Knight lab, Mayo 1-Mayo lab, Mayo 2, Bangladesh, and Colorado) with fecal samples collected using six different methods. SCCs are based on ten microbial composition metrics (panel A; centered log-ratio transformed abundance of four phyla, two alpha diversity metrics [number of observed OTUs and Shannon index] and four beta diversity metrics [unweighted UniFrac, generalized UniFrac, weighted UniFrac, and Bray-Curtis distance]), and centered log-ratio transformed select bacterial genera (panel B) with prevalence in the population >80% and a mean relative abundance >0.2%. *Abbreviations*: *BC*, *Bray-Curtis distance; FIT*, *fecal immunochemical test tubes; FOBT*, *fecal occult blood test cards; FTA*, *Flinders Technology Associates cards; GUniFrac*, *generalized UniFrac distance; SCC*, *Spearman correlation coefficient; UniFrac*, *unweighted UniFrac distance; WUnifrac distance*, *weighted UniFrac distance*.
(TIF)

S1 File Table A: Meta-analyzed ICCs^a^ (intraclass correlation coefficients) based on a random effects model for technical reproducibility comparing duplicate samples frozen day-0 from fecal samples collected using six different methods among 132 participants in five studies (Mayo 1-Knight lab, Mayo 1-Mayo lab, Mayo 2, Bangladesh, and Colorado); Table B: Meta-analyzed ICCs^a^ (intraclass correlation coefficients) based on random effects models for microbiome stability comparing fecal samples frozen on day-4/7 to those frozen at day-0 for six fecal sample collection methods among 132 participants in five studies (Mayo 1-Knight lab, Mayo 1-Mayo lab, Mayo 2, Bangladesh, and Colorado); Table C: Meta-analyzed log-fold changes based on random effects models for microbiome stability comparing fecal samples frozen on day-4/7 to those frozen at day-0 for six fecal sample collection methods among 132 participants in five studies (Mayo 1-Knight lab, Mayo 1-Mayo lab, Mayo 2, Bangladesh, and Colorado). Log-fold changes in relative abundance from day-0 are based on select taxa with prevalence > 50% and a mean read count > 10. Abbreviations: FIT, fecal immunochemical tests tubes; FOBT, fecal occult blood test cards; FTA, Flinders Technology Associates cards; Table D: Meta-analyzed SCCs (Spearman correlation coefficients) based on random effects model for accuracy of each collection method compared to no-solution samples frozen on day-0 (the gold standard) among 132 participants in five studies (Mayo 1-Knight lab, Mayo 1-Mayo lab, Mayo 2, Bangladesh, and Colorado) with fecal samples collected using six different methods; Table E: Meta-analyzed log-fold changes based on random effects models for accuracy of each collection method compared to no-solution samples frozen on day-0 (the gold standard) among 132 participants in five studies (Mayo 1-Knight lab, Mayo 1-Mayo lab, Mayo 2, Bangladesh, and Colorado). Log-fold changes in relative abundance from day-0 are based on select taxa with prevalence > 50% and a mean read count > 10. Abbreviations: FIT, fecal immunochemical tests tubes; FOBT, fecal occult blood test cards; FTA, Flinders Technology Associates cards.
(XLSX)

S1 Table Rationale for use, feasibility considerations, and previous findings for reproducibility, stability, and accuracy of six fecal sample collection methods for microbiome studies. (DOCX)

## References

[1] Wang X, Yang Y, Huycke MM. Microbiome-driven carcinogenesis in colorectal cancer: Models and mechanisms. Free Radic Biol Med. 2017;105:3–15. 10.1016/j.freeradbiomed.2016.10.504 27810411

[2] Kostic AD, Xavier RJ, Gevers D. The microbiome in inflammatory bowel disease: current status and the future ahead. Gastroenterology. 2014;146(6):1489–99. 10.1053/j.gastro.2014.02.009 24560869 PMC4034132

[3] Qin J, Li Y, Cai Z, Li S, Zhu J, Zhang F, et al A metagenome-wide association study of gut microbiota in type 2 diabetes. Nature. 2012;490(7418):55–60. 10.1038/nature11450 23023125

[4] Turnbaugh PJ, Ley RE, Mahowald MA, Magrini V, Mardis ER, Gordon JI. An obesity-associated gut microbiome with increased capacity for energy harvest. Nature. 2006;444(7122):1027–31. 10.1038/nature05414 17183312

[5] Choo JM, Leong LE, Rogers GB. Sample storage conditions significantly influence faecal microbiome profiles. Scientific Reports. 2015;5:16350 10.1038/srep16350 26572876 PMC4648095

[6] Vandeputte D, Tito RY, Vanleeuwen R, Falony G, Raes J. Practical considerations for large-scale gut microbiome studies. FEMS Microbiol Rev. 2017;41(Supp_1):S154–S67. 10.1093/femsre/fux027 28830090 PMC7207147

[7] Dominianni C, Wu J, Hayes RB, Ahn J. Comparison of methods for fecal microbiome biospecimen collection. BMC Microbiology. 2014;14:103 10.1186/1471-2180-14-103 24758293 PMC4005852

[8] Bundgaard-Nielsen C, Hagstrøm S, Sørensen S. Interpersonal Variations in Gut Microbiota Profiles Supersedes the Effects of Differing Fecal Storage Conditions. Scientific Reports. 2018(June):1–9. 10.1038/s41598-017-17765-5 30478355 PMC6255890

[9] Roesch LF, Casella G, Simell O, Krischer J, Wasserfall CH, Schatz D, et al Influence of fecal sample storage on bacterial community diversity. Open Microbiol J. 2009;3:40–6. 10.2174/1874285800903010040 19440250 PMC2681173

[10] Fu BC, Randolph TW, Lim U, Monroe KR, Cheng I, Wilkens LR, et al Characterization of the gut microbiome in epidemiologic studies: the multiethnic cohort experience. Annals of Epidemiology. 2016;26(5):373–9. 10.1016/j.annepidem.2016.02.009 27039047 PMC4892953

[11] McInnes P, Cutting M. Manual of Procedures for Human Microbiome Project, V 12.0. 2010.

[12] Sinha R, Chen J, Amir A, Vogtmann E, Shi J, Inman KS, et al Collecting fecal samples for microbiome analyses in epidemiology studies. Cancer Epidemiology Biomarkers & Prevention. 2016;25(2):407–16. 10.1158/1055-9965.EPI-15-0951 PMC4821594 26604270

[13] Song SJ, Amir A, Metcalf JL, Amato KR, Xu ZZ, Humphrey G, et al Preservation Methods Differ in Fecal Microbiome Stability, Affecting Suitability for Field Studies. mSystems. 2016;1(3):e00021–16. 10.1128/mSystems.00021-16 27822526 PMC5069758

[14] Vogtmann E, Chen J, Amir A, Shi J, Abnet CC, Nelson H, et al Comparison of Collection Methods for Fecal Samples in Microbiome Studies. Am J Epidemiol. 2017;185(2):115–23. 10.1093/aje/kww177 27986704 PMC5253972

[15] Vogtmann E, Chen J, Kibriya MG, Chen Y, Islam T, Eunes M, et al Comparison of Fecal Collection Methods for Microbiota Studies in Bangladesh. Appl Environ Microbiol. 2017;83(10). 10.1128/AEM.00361-17 PMC5411505 28258145

[16] Sinha R, Chen J, Amir A, Vogtmann E, Shi J, Inman KS, et al Collecting Fecal Samples for Microbiome Analyses in Epidemiology Studies. Cancer Epidemiol Biomarkers Prev. 2016;25(2):407–16. 10.1158/1055-9965.EPI-15-0951 26604270 PMC4821594

[17] Caporaso JG, Lauber CL, Walters WA, Berg-Lyons D, Huntley J, Fierer N, et al Ultra-high-throughput microbial community analysis on the Illumina HiSeq and MiSeq platforms. The ISME Journal. 2012;6(8):1621–4. 10.1038/ismej.2012.8 22402401 PMC3400413

[18] Walters WA, Caporaso JG, Lauber CL, Berg-Lyons D, Fierer N, Knight R. PrimerProspector: de novo design and taxonomic analysis of barcoded polymerase chain reaction primers. Bioinformatics. 2011;27(8):1159–61. 10.1093/bioinformatics/btr087 21349862 PMC3072552

[19] Caporaso JG, Kuczynski J, Stombaugh J, Bittinger K, Bushman FD, Costello EK, et al QIIME allows analysis of high-throughput community sequencing data. Nature Methods. 2010;7(5):335–6. 10.1038/nmeth.f.303 20383131 PMC3156573

[20] Amir A, McDonald D, Navas-Molina JA, Kopylova E, Morton JT, Zech Xu Z, et al Deblur Rapidly Resolves Single-Nucleotide Community Sequence Patterns. mSystems. 2017;2(2):e00191–16. 10.1128/mSystems.00191-16 28289731 PMC5340863

[21] McMurdie PJ, Holmes S. phyloseq: an R package for reproducible interactive analysis and graphics of microbiome census data. PloS One. 2013;8(4):e61217 10.1371/journal.pone.0061217 23630581 PMC3632530

[22] Dixon P. VEGAN, a package of R functions for community ecology. Journal of Vegetation Science. 2003;14(6):927–30.

[23] Chen J. Package 'GUniFrac'. 2018.

[24] Rosner B. Fundamentals of biostatistics. 8th edition ed. Boston, MA: Cengage Learning; 2016 xix, 927.

[25] Gloor GB, Wu JR, Pawlowsky-Glahn V, Egozcue JJ. It's all relative: analyzing microbiome data as compositions. Ann Epidemiol. 2016;26(5):322–9. 10.1016/j.annepidem.2016.03.003 27143475

[26] Kostic AD, Chun E, Robertson L, Glickman JN, Gallini CA, Michaud M, et al Fusobacterium nucleatum potentiates intestinal tumorigenesis and modulates the tumor-immune microenvironment. Cell Host Microbe. 2013;14(2):207–15. 10.1016/j.chom.2013.07.007 23954159 PMC3772512

[27] Harrison XA. Using observation-level random effects to model overdispersion in count data in ecology and evolution. PeerJ. 2014;2:e616 10.7717/peerj.616 25320683 PMC4194460

[28] DerSimonian R, Kacker R. Random-effects model for meta-analysis of clinical trials: an update. Contemp Clin Trials. 2007;28(2):105–14. 10.1016/j.cct.2006.04.004 16807131

[29] Higgins JP, Thompson SG. Quantifying heterogeneity in a meta-analysis. Stat Med. 2002;21(11):1539–58. 10.1002/sim.1186 12111919

[30] Lauber CL, Zhou N, Gordon JI, Knight R, Fierer N. Effect of storage conditions on the assessment of bacterial community structure in soil and human-associated samples. FEMS Microbiol Lett. 2010;307(1):80–6. 10.1111/j.1574-6968.2010.01965.x 20412303 PMC3148093

[31] Carroll IM, Ringel-Kulka T, Siddle JP, Klaenhammer TR, Ringel Y. Characterization of the fecal microbiota using high-throughput sequencing reveals a stable microbial community during storage. PLoS One. 2012;7(10):e46953 10.1371/journal.pone.0046953 23071673 PMC3465312

[32] Shaw AG, Sim K, Powell E, Cornwell E, Cramer T, McClure ZE, et al Latitude in sample handling and storage for infant faecal microbiota studies: the elephant in the room? Microbiome. 2016: 4(1):40 10.1186/s40168-016-0186-x 27473284 PMC4967342

[33] Stearns JC, Lynch MDJ, Senadheera DB, Tenenbaum HC, Goldberg MB, Cvitkovitch DG, et al Bacterial biogeography of the human digestive tract. Scientific Reports. 2011;1:170 10.1038/srep00170 22355685 PMC3240969

[34] Ott SJ, Musfeldt M, Timmis KN, Hampe J, Wenderoth DF, Schreiber S. In vitro alterations of intestinal bacterial microbiota in fecal samples during storage. Diagn Microbiol Infect Dis. 2004;50(4):237–45. 10.1016/j.diagmicrobio.2004.08.012 15582296

[35] Cardona S, Eck A, Cassellas M, Gallart M, Alastrue C, Dore J, et al Storage conditions of intestinal microbiota matter in metagenomic analysis. BMC Microbiol. 2012;12:158 10.1186/1471-2180-12-158 22846661 PMC3489833

[36] Amir A, McDonald D, Navas-Molina JA, Debelius J, Morton JT, Hyde E, et al Correcting for Microbial Blooms in Fecal Samples during Room-Temperature Shipping. mSystems. 2017;2(2): e00199–16. 10.1128/mSystems.00199-16 28289733 PMC5340865

[37] Baxter NT, Koumpouras CC, Rogers MA, Ruffin MTt, Schloss PD. DNA from fecal immunochemical test can replace stool for detection of colonic lesions using a microbiota-based model. Microbiome. 2016;4(1):59 10.1186/s40168-016-0205-y 27842559 PMC5109736

[38] Gudra D, Shoaie S, Fridmanis D, Klovins J, Wefer H, Silamikelis I, et al A widely used sampling device in colorectal cancer screening programmes allows for large-scale microbiome studies. Gut. 2018;68(9):1723–25 10.1136/gutjnl-2018-316225 30242040 PMC6709769

[39] Rounge TB, Meisal R, Nordby JI, Ambur OH, de Lange T, Hoff G. Evaluating gut microbiota profiles from archived fecal samples. BMC Gastroenterol. 2018;18(1):171 10.1186/s12876-018-0896-6 30409123 PMC6225565

[40] Nechvatal JM, Ram JL, Basson MD, Namprachan P, Niec SR, Badsha KZ, et al Fecal collection, ambient preservation, and DNA extraction for PCR amplification of bacterial and human markers from human feces. J Microbiol Methods. 2008;72(2):124–32. 10.1016/j.mimet.2007.11.007 18162191

[41] Taylor MW. Examining the potential use and long-term stability of guaiac faecal occult blood test cards for microbial DNA 16S rRNA sequencing. J Clin Pathol. 2017;70(7):600–6. 10.1136/jclinpath-2016-204165 28011577

[42] Tap J, Cools-Portier S, Pavan S, Druesne A, Öhman L, Törnblom H, et al Effects of the long-term storage of human fecal microbiota samples collected in RNAlater. Scientific Reports. 2019;9(1):1–9. 10.1038/s41598-018-37186-2 30679604 PMC6345939

[43] Wang Z, Zolnik CP, Qiu Y, Usyk M, Wang T, Strickler HD, et al Comparison of Fecal Collection Methods for Microbiome and Metabolomics Studies. Frontiers in Cellular and Infection Microbiology. 2018;8(August):1–10. 10.3389/fcimb.2018.00001 30234027 PMC6127643

[44] Hale VL, Tan CL, Knight R, Amato KR. Effect of preservation method on spider monkey (Ateles geoffroyi) fecal microbiota over 8 weeks. J Microbiol Methods. 2015;113:16–26. 10.1016/j.mimet.2015.03.021 25819008

[45] Vlckova K, Mrazek J, Kopecny J, Petrzelkova KJ. Evaluation of different storage methods to characterize the fecal bacterial communities of captive western lowland gorillas (Gorilla gorilla gorilla). J Microbiol Methods. 2012;91(1):45–51. 10.1016/j.mimet.2012.07.015 22828127

[46] Kilpatrick CW. Noncryogenic preservation of mammalian tissues for DNA extraction: an assessment of storage methods. Biochem Genet. 2002;40(1–2):53–62. 10.1023/a:1014541222816 11989787

[47] Franzosa EA, Morgan XC, Segata N, Waldron L, Reyes J, Earl AM, et al Relating the metatranscriptome and metagenome of the human gut. Proc Natl Acad Sci U S A. 2014;111(22):E2329–38. 10.1073/pnas.1319284111 24843156 PMC4050606

[48] Gorzelak MA, Gill SK, Tasnim N, Ahmadi-Vand Z, Jay M, Gibson DL. Methods for Improving Human Gut Microbiome Data by Reducing Variability through Sample Processing and Storage of Stool. PLoS One. 2015;10(8):e0134802 10.1371/journal.pone.0134802 26252519 PMC4529225

[49] Voigt AY, Costea PI, Kultima JR, Li SS, Zeller G, Sunagawa S, et al Temporal and technical variability of human gut metagenomes. Genome Biol. 2015;16:73 10.1186/s13059-015-0639-8 25888008 PMC4416267

[50] Chen Z, Hui PC, Hui M, Yeoh YK, Wong PY, Chan MCW, et al Impact of Preservation Method and 16S rRNA Hypervariable Region on Gut Microbiota Profiling. mSystems. 2019;4(1):1–15. 10.1128/mSystems.00271-18 PMC6392095 30834331

[51] Flores R, Shi J, Yu G, Ma B, Ravel J, Goedert JJ, et al Collection media and delayed freezing effects on microbial composition of human stool. Microbiome. 2015;3:33 10.1186/s40168-015-0092-7 26269741 PMC4534027

[52] Shah MS, DeSantis TZ, Weinmaier T, McMurdie PJ, Cope JL, Altrichter A, et al Leveraging sequence-based faecal microbial community survey data to identify a composite biomarker for colorectal cancer. Gut. 2018;67(5):882–91. 10.1136/gutjnl-2016-313189 28341746

[53] Drew DA, Lochhead P, Abu-Ali G, Chan AT, Huttenhower C, Izard J. Fecal microbiome in epidemiologic studies—letter. Cancer Epidemiology Biomarkers & Prevention. 2016: 25(5):869. 10.1158/1055-9965.EPI-16-0063 PMC4898650 26961995

[54] Sinha R, Vogtmann E, Chen J, Amir A, Shi J, Sampson J, et al Fecal Microbiome in Epidemiologic Studies—Response. Cancer Epidemiology Biomarkers & Prevention. 2016;25(5):870–1. 10.1158/1055-9965.EPI-16-0161 PMC4873434 26961994

[55] Loftfield E, Vogtmann E, Sampson JN, Moore SC, Nelson H, Knight R, et al Comparison of collection methods for fecal samples for discovery metabolomics in epidemiological studies. Cancer Epidemiology Biomarkers & Prevention. 2016;25(11):1483–90. 10.1158/1055-9965.EPI-16-0409 PMC5093035 27543620

