# Supplementary figures and images for: Reproducibility, stability, and accuracy of microbial profiles by fecal sample collection method in three distinct populations
Source: PLoS One. 2019 Nov 18;14(11):e0224757. doi: 10.1371/journal.pone.0224757 (PMC6860998; doi:10.1371/journal.pone.0224757)

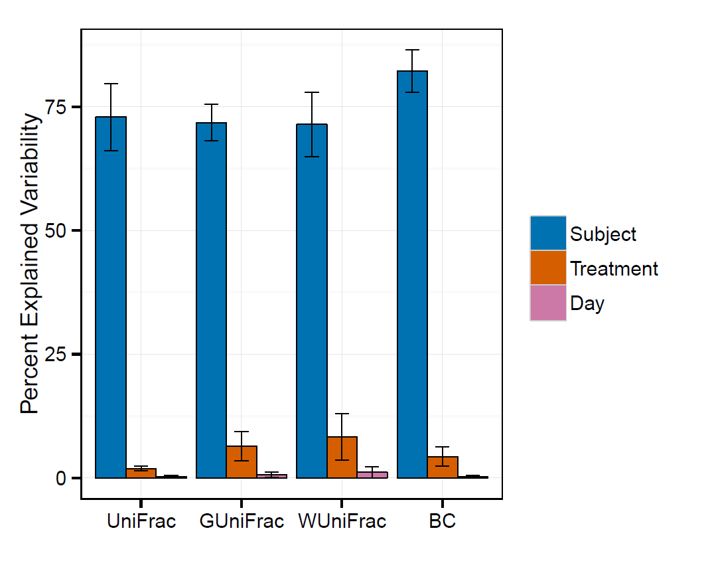

Supplement: S1 Fig — Error bars represent the standard deviation across all the studies. Abbreviations: UniFrac, unweighted UniFrac; gUniFrac, generalized UniFrac; wUniFrac, weighted UniFrac; BC, Bray-Curtis distance. (TIF) [file pone.0224757.s001.tif]

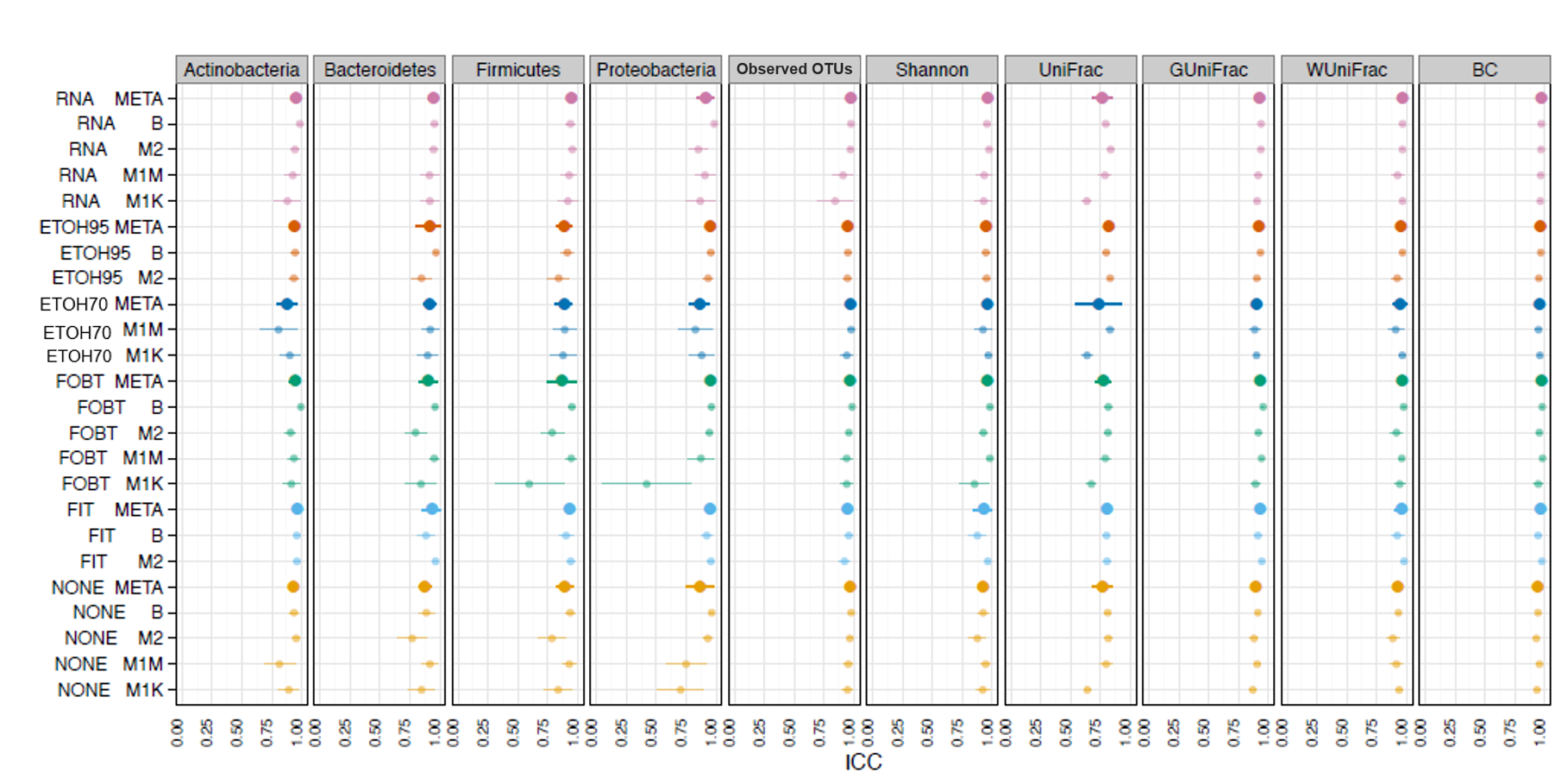

Supplement: S2 Fig — Abbreviations: B, Bangladesh; FIT, fecal immunochemical tests; FOBT, fecal occult blood test cards; FTA. Flinders Technology Associates (FTA) cards; M2, Mayo Clinic 2 study; M1M, Mayo Clinic 1 study, Mayo Clinic lab; M1K, Mayo Clinic 1 study, Knight lab; META, meta-analysis (pooled samples). (TIF) [file pone.0224757.s002.tif]

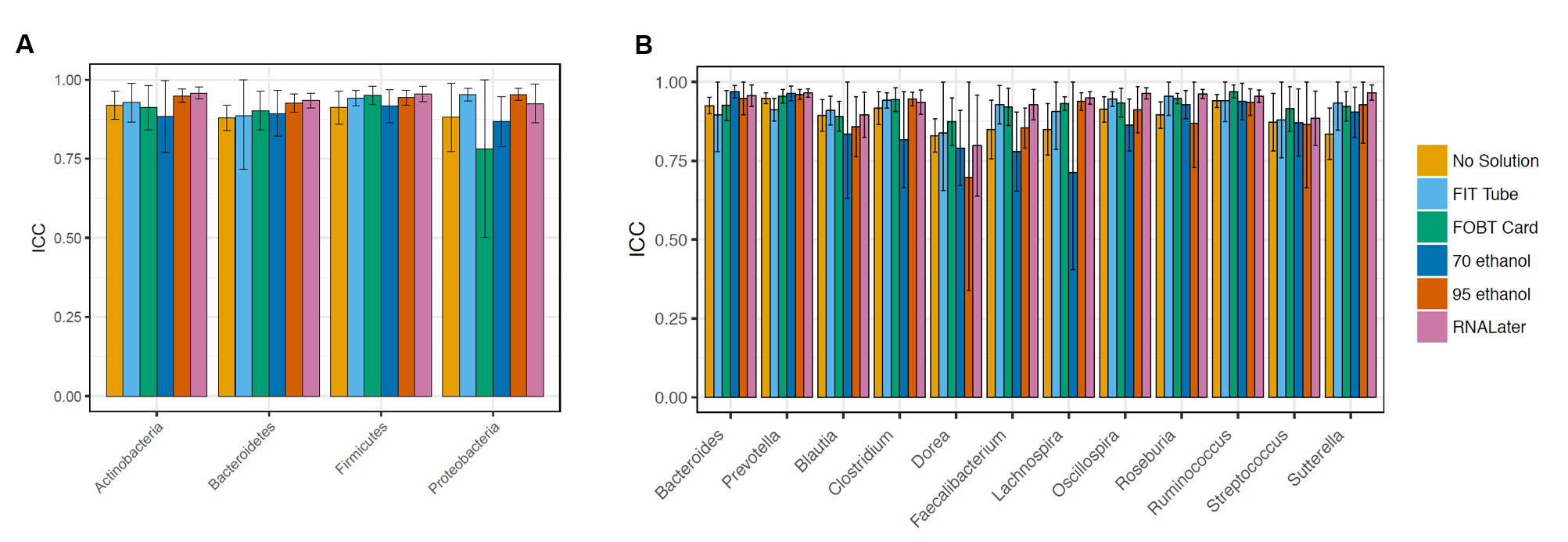

Supplement: S3 Fig — ICCs are based on ten microbial composition metrics (panel A; centered log-ratio transformed abundance of four phyla, two alpha diversity metrics [number of observed OTUs and Shannon index] and four beta diversity metrics [unweighted UniFrac, generalized UniFrac, weighted UniFrac, and Bray-Curtis distance]), and centered log-ratio transformed select bacterial genera (panel B) with prevalence in the population >80% and a mean relative abundance >0.2%. Error bars represent 95% CIs. Abbreviations: BC, Bray-Curtis distance; FIT, fecal immunochemical test tubes; FOBT, fecal occult blood test cards; FTA, Flinders Technology Associates cards; GUniFrac, generalized UniFrac distance; ICC, intraclass correlation coefficient; UniFrac, unweighted UniFrac distance; WUnifrac, weighted UniFrac distance. (TIF) [file pone.0224757.s003.tif]

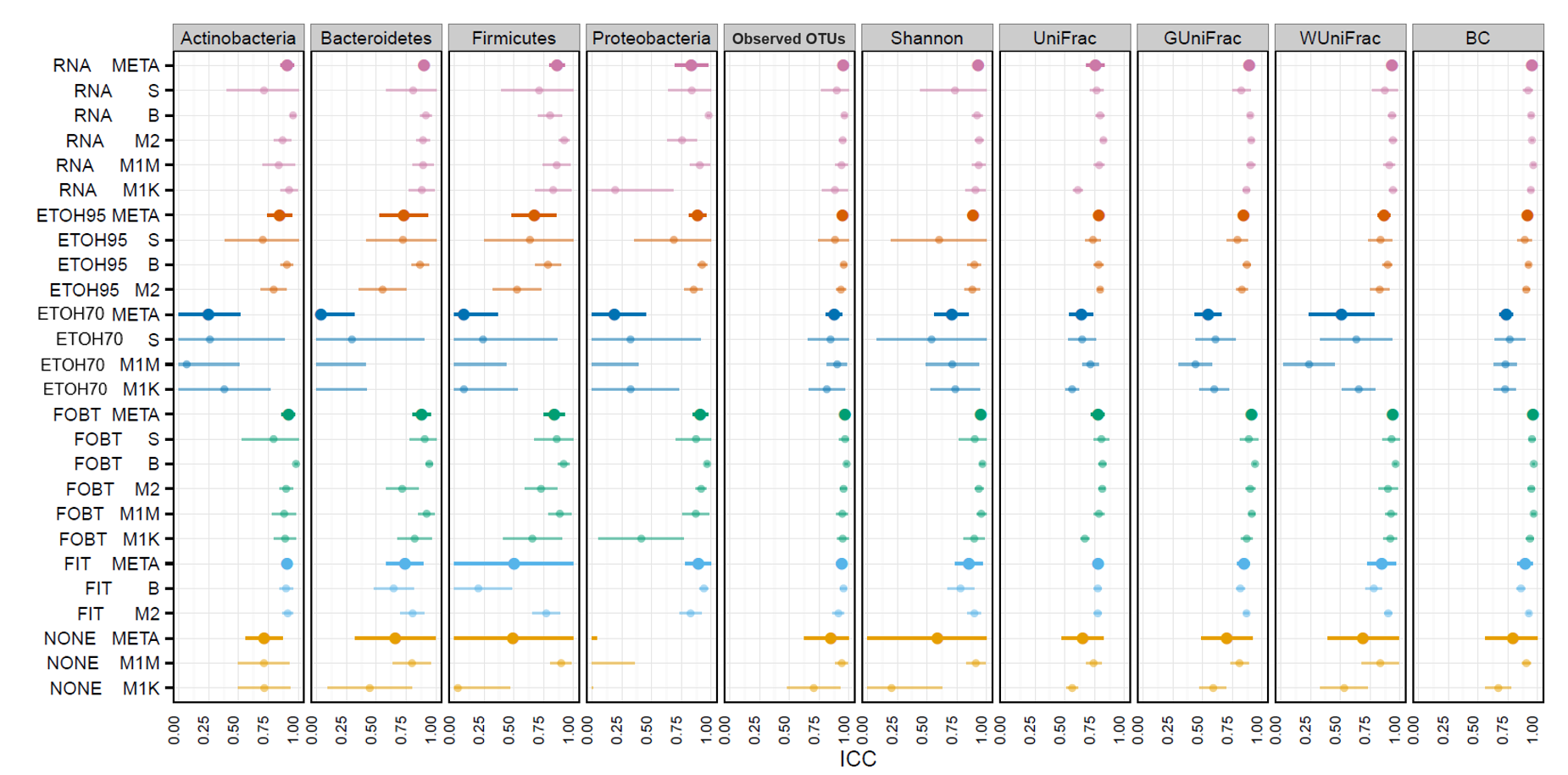

Supplement: S4 Fig — Abbreviations: B, Bangladesh; FIT, fecal immunochemical tests; FOBT, fecal occult blood test cards; FTA. Flinders Technology Associates (FTA) cards; M2, Mayo Clinic 2 study; M1M, Mayo Clinic 1 study, Mayo Clinic lab; M1K, Mayo Clinic 1 study, Knight lab; S, Colorado study; META, meta-analysis (pooled samples). (TIF) [file pone.0224757.s004.tif]

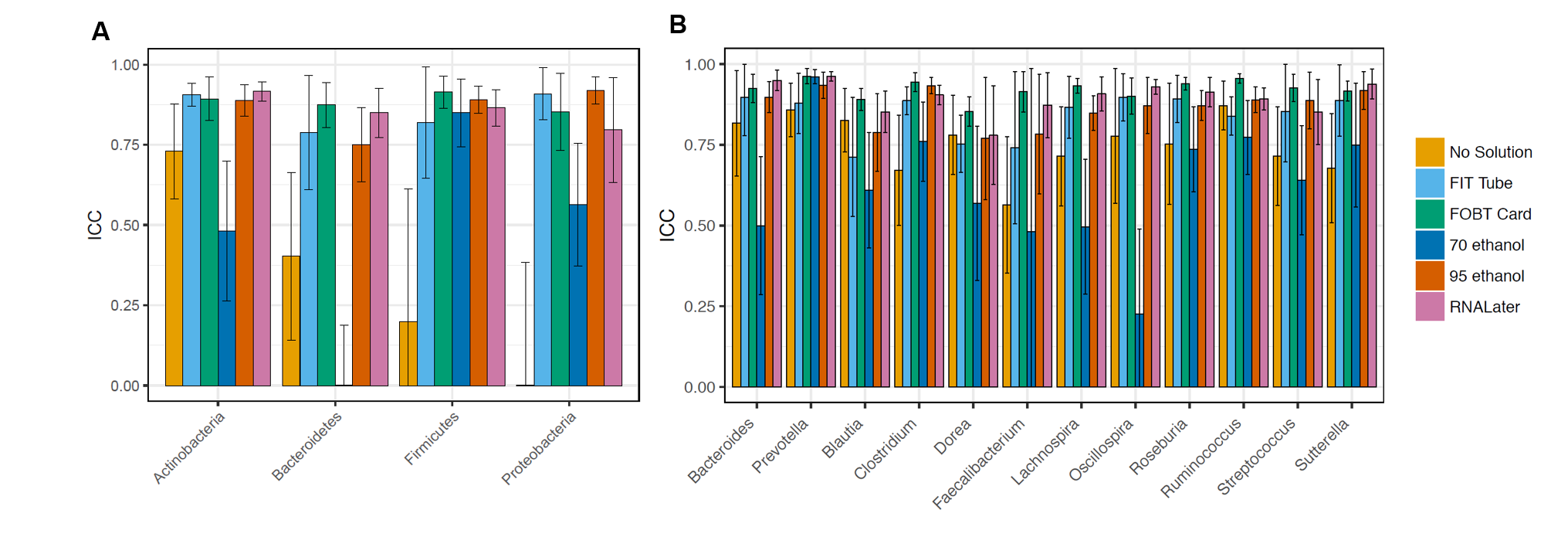

Supplement: S5 Fig — ICCs are based on ten microbial composition metrics (panel A; centered log-ratio transformed abundance of four phyla, two alpha diversity metrics [number of observed OTUs and Shannon index] and four beta diversity metrics [unweighted UniFrac, generalized UniFrac, weighted UniFrac, and Bray-Curtis distance]), and centered log-ratio transformed select bacterial genera (panel B) with prevalence in the population >80% and a mean relative abundance >0.2%. All error bars represent 95% CIs. Abbreviations: BC, Bray-Curtis distance; FIT, fecal immunochemical tests tubes; FOBT, fecal occult blood test cards; FTA, Flinders Technology Associates cards; GUniFrac, generalized UniFrac distance; ICC, intraclass correlation coefficient; UniFrac, unweighted UniFrac distance; WUnifrac, weighted UniFrac distance. (TIF) [file pone.0224757.s005.tif]

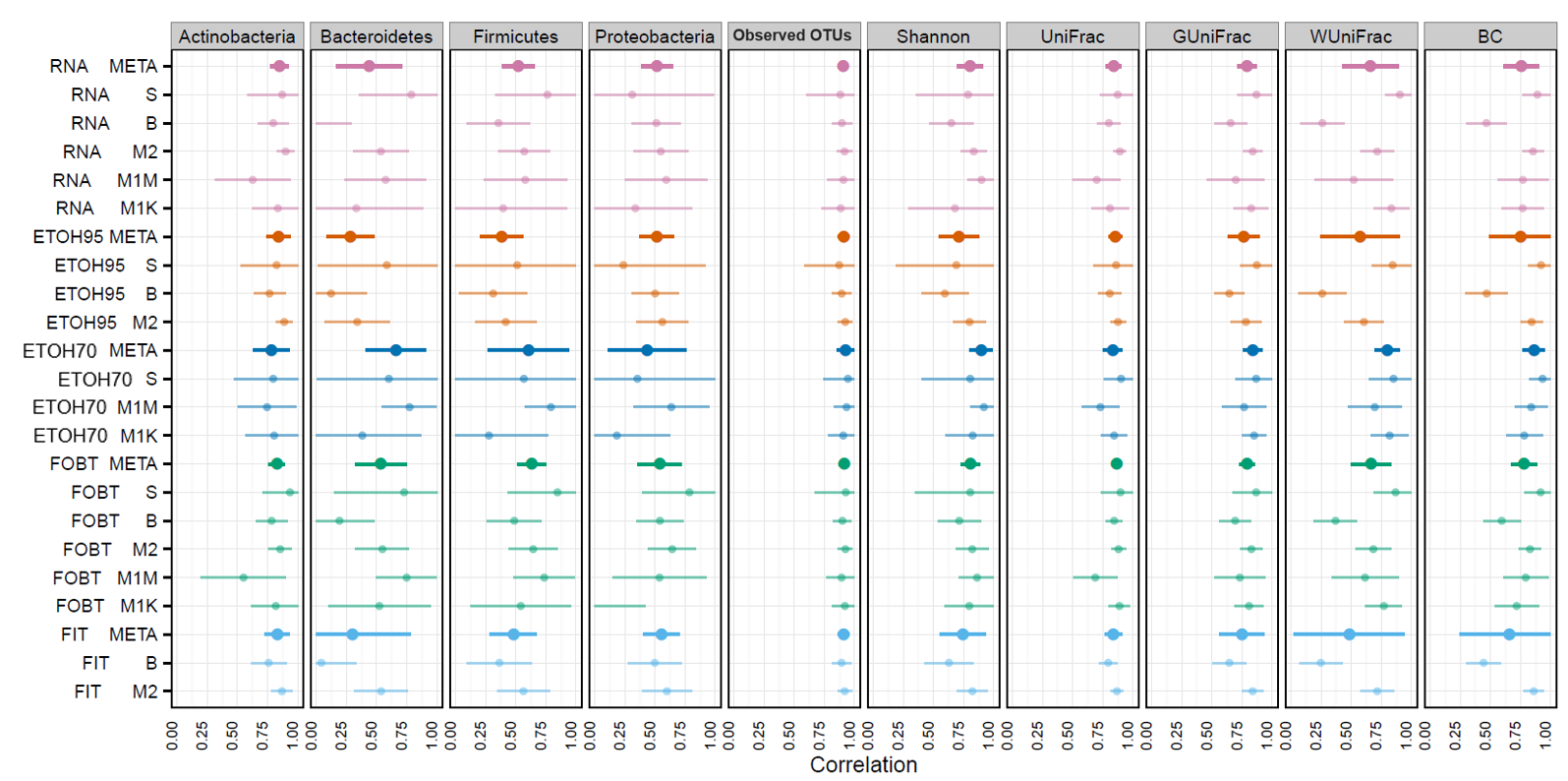

Supplement: S6 Fig — Samples at T = 0 were compared with flash-frozen samples. Abbreviations: B, Bangladesh study; FIT, fecal immunochemical tests; FOBT, fecal occult blood test cards; FTA. Flinders Technology Associates (FTA) cards; M2, Mayo Clinic 2 study; M1M, Mayo Clinic 1 study, Mayo Clinic lab; M1K, Mayo Clinic 1 study, Knight lab; S, Colorado study; META, meta-analysis (pooled samples). (TIF) [file pone.0224757.s006.tif]

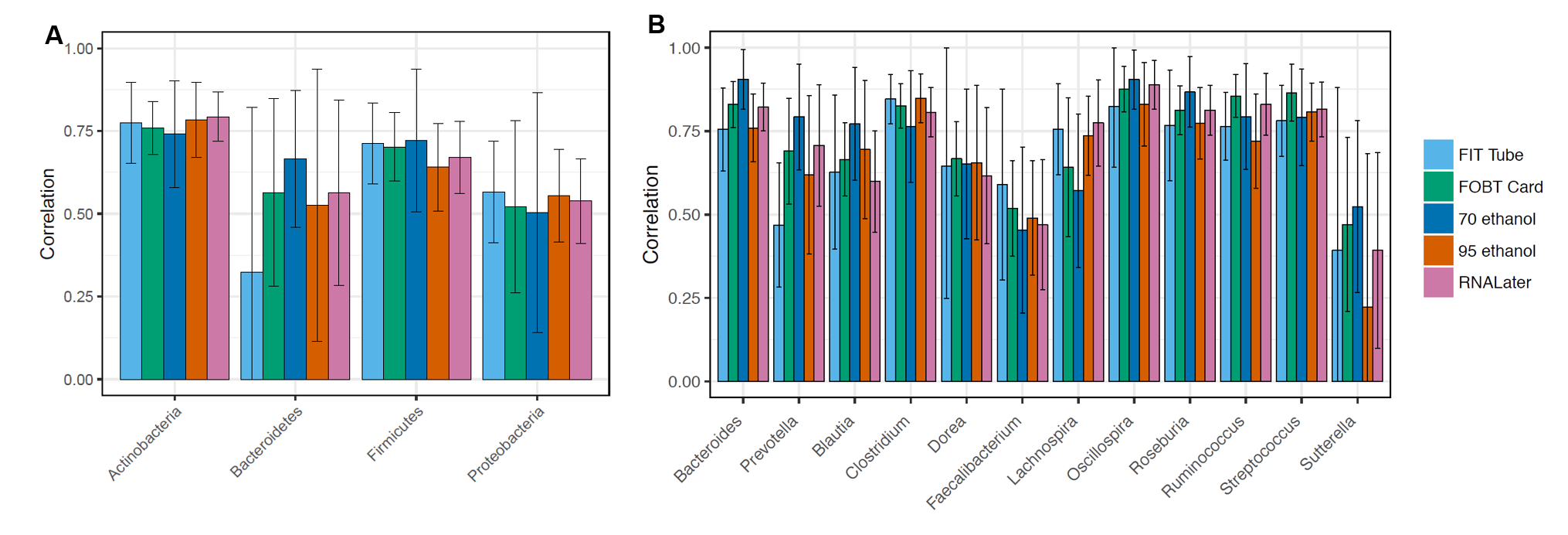

Supplement: S7 Fig — SCCs are based on ten microbial composition metrics (panel A; centered log-ratio transformed abundance of four phyla, two alpha diversity metrics [number of observed OTUs and Shannon index] and four beta diversity metrics [unweighted UniFrac, generalized UniFrac, weighted UniFrac, and Bray-Curtis distance]), and centered log-ratio transformed select bacterial genera (panel B) with prevalence in the population >80% and a mean relative abundance >0.2%. Abbreviations: BC, Bray-Curtis distance; FIT, fecal immunochemical test tubes; FOBT, fecal occult blood test cards; FTA, Flinders Technology Associates cards; GUniFrac, generalized UniFrac distance; SCC, Spearman correlation coefficient; UniFrac, unweighted UniFrac distance; WUnifrac distance, weighted UniFrac distance. (TIF) [file pone.0224757.s007.tif]
