# Supplementary material for: Reproducibility, stability, and accuracy of microbial profiles by fecal sample collection method in three distinct populations
Source: PLoS One. 2019 Nov 18;14(11):e0224757. doi: 10.1371/journal.pone.0224757 (PMC6860998; doi:10.1371/journal.pone.0224757)
Supplement: S1 Table — (DOCX) [file pone.0224757.s009.docx]

**S6 Table.** Rationale for use, feasibility considerations, and previous findings for reproducibility, stability, and accuracy of six fecal sample collection methods for microbiome studies

| **Collection method, their rationale for use, and their feasibility considerations** | **Summary of previous findings for reproducibility, stability, and accuracy** |
| --- | --- |
| **No solution**  *Rationale for use*  Thought to be the “gold standard” preservation method if immediately-frozen at -80°C, as these samples likely reflect those microbes undisturbed from their original environment  *Feasibility considerations*  Immediately freezing samples at -80°C is often infeasible in larger, field-based studies; no DNA stabilizing properties when left at room temperature for longer periods of time | Relative abundances:  ↑ *Actinobacteria* after 2w at RT (infant samples) [1]  ≈ *Actinobacteria* after 1h, 4h, 6h, 8h, and 24h at RT [2]  ↓ *Actinobacteria* after 72h at RT [3]  ≈ *Bacteroides* after 1h, 4h, 6h, 8h, 24h at RT [2], and ≈ *Bacteroides_Bacteroidaceae, Bacteroides_Porphyromonadaceae, Bacteroides_Rikenellaceae* after 3d or 14d at 20°C [4]  ↓ *Bacteroides* after 12h, 24h, 48h, 72h [5], and 2w [6] at RT  ↓ *Bifidobacterium* after 72h at RT [3]  ≈ *Bifidobacterium* after 3h, 24h at RT [6]  ↓ *Clostridium* after 12h, 24h, 48h, and 72h at RT [5]  ↑ *Enterobacteriaceae* after 12h, 24h, 48h, and 72h at RT [5]  ↓ *Firmicutes* after 2w (infant samples) [1] and 72h at RT [3]  ≈ *Firmicutes* after 1h, 4h, 6h, 8h, 24h, [2] and 72h [7] at RT, and ≈ *Firmicutes Lachnospiraceae, Firmicutes* *Ruminococcaceae,* and *Firmicutes* *Veillonellaceae* after 3d or 14d stored at 20°C [4]  ≈ *Prevotellaceae* after 3h and 24h at RT [6]  ↑ *Proteobacteria* after 2w at RT (infant samples) [1]  ≈ *Proteobacteria* after 1h, 4h, 6h, 8h, and 24h at RT [2]  ↓ *Veillonella* after 1w-2w at RT (infant samples) [1]  α diversity:  ≈ OTU richness after 12-28h at 4°C and 1-2 thaw cycles at -20°C vs. GS [8], and after 72h at 4°C and RT vs. GS [3]  ↓ bacterial diversity and bacterial counts after 8-24h at RT [9, 10]  ↓ Shannon Index after ≤2w at RT (infant samples) [1] and after 72h at RT [3];  ≈ Shannon Index after 15-30min, 1-2h [11], 48h[12], 72h [7] at RT, 24h at4°C and RT [13]  ≈ Inverse Simpson Index after 48h at RT [11]  ≈ Chao1 Index after 15-30min and 1-2h at RT [11], and 24h at 4°C and RT [13]  ≈ Observed OTUs after 15-30min and 1-2h at RT [11], and after 3h, 24h, 48h, 72h, and 2w at RT [6, 12];  ≈ PD Whole Tree diversity after 24h at 4°C and RT [13]  β diversity:  *↑* Unweighted UniFrac distances from immediately-frozen samples after 1w (the latter in infant samples) [1]  Did not cluster based on Unweighted or Weighted UniFrac distance from immediately-frozen samples after 15-30min and 1-2h at RT [11], after 24h at 4°C and RT [13], and after 48 hours on ice [14]  *↑* Weighted UniFrac distances from immediately-frozen samples after 12h and 48h (RT) (infant samples) [1],  *↑* Weighted UniFrac distances from immediately-frozen samples after 24h at 4°C and RT [13], and after at 3h and 24h at RT [6]  *↑* Bray-Curtis pairwise distances from immediately-frozen samples after 24h at RT (infant samples) [1];  Did not cluster based on Bray-Curtis pairwise distances from immediately-frozen samples after 24h at 4°C and RT [13] and after 48 hours on ice [14] |
| **FIT tube**  *Rationale*  Stabilize DNA via antimicrobial agents; used in settings for colorectal cancer screening, which may open opportunities for the establishment of prospective cohorts  *Feasibility considerations*  Previously found to be suboptimal for metabolomics studies (especially when stored at ambient temperature) [15]; stabilizing solutions are a trade-secret and may differ by manufacturer [16] | Relative abundances:  ↓ *Actinobacteria* after 48h at 4°C compared to GS (immediately frozen, no-solution samples) [17]  *↑* detection of genus *Pantoea* and *Helicobacter* compared to GS [17]  α diversity:  Slightly ↓ Shannon Diversity after 1d-2d and 7d (at 4°C, 20°C, 30°C), and 14d (at 4°C, 20°C) with ↑ storage time vs. GS and immediately-frozen FIT samples [16]  ≈ Inverse Simpson and Shannon Diversity comparing FIT cards at RT for 24h to GS [12]  ↓ number of OTUs for immediately-frozen FIT vs. GS [12]  β diversity:  *↑* Weighted UniFrac distances from immediately-frozen no-solution and immediately-frozen FIT samples with ↑ storage time ranging 1d-14d (at 4°C, 20°C, 30°C) compared to [16]  Microbiota composition differed based on Bray-Curtis distances for immediately-frozen FIT vs. GS (*p*<0.001) [12] |
| **FTA/FOBT card**  *Feasibility considerations*  Optimal for metabolomics studies [15]; used in settings for colorectal cancer screening, which open many opportunities for the establishment of prospective cohorts | ≈ DNA preservation after 2w-12w (vs. immediately frozen FOBT cards) and 7m-36m at RT [18], and after 5d at RT (the latter vs. GS) [19]  ↓ DNA concentration on FTA cards after 8wks at RT in monkey fecal samples [20]  Relative abundances:  ICC = 0.97 for Actinobacteria comparing FTA cards at RT for 24h to GS [21]  ICC = 0.91 for Firmicutes comparing FTA cards at RT for 24h to GS [21]  ≈ Bacteroidetes, Firmicutes, and Actinobacteria for FOBT card after 72h at RT vs. GS [7]  α diversity:  ICC = 0.96, 0.96, 0.76 for Shannon index, Simpson’s Index, Chao-1 Index, respectively comparing FTA cards at RT for 24h to GS [21]  ≈ Shannon Index after 72h at RT compared to GS [7] |
| **95% ethanol**  *Rationale*  Ethanol has anti-microbial properties and stabilizes bacterial DNA  *Feasibility considerations*  Considerably cheaper than other solutions, such as RNAlater; performs well in metabolomics and metatranscriptomics studies [22, 23]; has volatile properties (e.g., is flammable), and thus may be more expensive to ship | ↓ DNA yields over time in tissue samples [24] and gorilla fecal samples (latter in 96% ethanol) [25]  ≈ DNA concentration in 100% ethanol after 8wks at RT in monkey fecal samples [20]  Relative abundances:  ICC = 0.97 for Actinobacteria stored at RT for 24h vs.GS [21]  ICC = 0.34 for Firmicutes stored at RT for 24h vs. GS [21]  ≈ Bacteroides counts for immediately frozen 96% ethanol samples vs. GS [25]  ≈ Enterobacteriaceae counts for immediately frozen 96% ethanol samples vs. GS [25]  α diversity:  ICCs = 0.25, 0.36, 0.01 for Shannon index, Simpson’s Index, Chao-1 Index, respectively, stored at RT for 24h vs. GS [21] |
| **70% ethanol**  *Rationale*  See description above  *Feasibility considerations*  See description above; additionally, dilution may lead to higher DNA degradation over time | No previous studies available |
| **RNA*later***  *Rationale*  Acts as both DNA and RNA stabilizer by preventing degradation by nucleases when specimens thawed before extraction [26]  *Feasibility considerations*  Preserves sufficient DNA for 16S and metagenomic analyses, as well as transcriptomics, but not metabolomics due to high sodium sulfate content [27]; a relatively expensive method as it requires larger volume ratios of sample to RNA*later*; has associated storage costs | Overall findings:  ↓ DNA purity after storage at RT for 72h vs. GS[7]  ≈ DNA yield after storage at RT for 5d [19], and vs. GS (gorilla fecal samples) [25]  Excellent ICCs (>75%) for reproducibility of all phyla, α diversity, and β diversity metrics [26]  Relative abundances:  ICC = 0.84 for Actinobacteria after storage at RT for 24h vs. GS [21]  ICC = 0.64 for Firmicutes after storage at RT for 24h vs. GS [21]  ≈ Bacteroides counts for immediately frozen RNA*later* samples vs. GS [25]  ↑ Bacteroides after storage at RT for 72h [3], and when stored at RT for 7d [28]  ↑ Bacteroidetes for immediately frozen RNA*later* samples vs. GS (also had higher reproducibility ICC for this phylum than GS) [26]  ≈ Bacteroidetes after storage at RT for 72h vs. GS [7]  ↑ Ruminococcaceae after storage at RT for 7d [28]  ↓ Firmicutes after storage at RT for 72h vs. GS [3]  ≈ Firmicutes after storage at RT for 72h vs. GS [7]  ↓ Actinobacteria after storage at RT for 72h vs. GS [3]  ≈ Actinobacteria after storage at RT for 72h vs. GS [7]  ≈ Enterobacteriaceae counts for immediately frozen RNA*later* samples vs. GS [25]  Stability ICCs=0.96, 0.76, 0.73, 0.60 for Actinobacteria, Bacteroidetes, Firmicutes, and Proteobacteria, respectively, after 3d storage at RT[26]  Stability ICCs=0.98, 0.70, 0.68, 0.64 for Actinobacteria, Bacteroidetes, Firmicutes, and Proteobacteria, respectively, after 7d storage at RT[26]  α diversity:  ↓ Shannon Diversity after storage at RT for 72h [7], and after storage at 4-10°C for 1w [29]  ICC = 0.75, 0.79, 0.51 for Shannon index, Simpson’s Index, Chao-1 Index, respectively, after storage at RT for 24h vs. GS [21]  ≈ observed OTUs after storage at RT for 72h [3], or at 4-10°C for 1w [29]  ≈ observed OTUs and evenness (Inverse Shannon Index) for immediately-frozen RNA*later* samples vs. GS [30]  ↓ OTU evenness after storage at RT for 72h [3], or at RT or 4-10°C for 1w [29]  ↓ OTU richness for immediately-frozen RNA*later* samples vs. GS [25, 31]  Stability ICCs=0.40, 0.53, 0.77 for Chao1, observed species, and Shannon Index, respectively, after 3d storage at RT [26]  Stability ICCs=0.76, 0.78, 0.88 for Chao1, observed species, and Shannon Index, respectively, after 7d storage at RT [26]  β diversity:  Generally excellent stability ICCs (>75%) for β diversity (weighted and unweighted UniFrac) after 3d or 7d storage at RT [26] |

Key: GS, gold standard (fecal sample with no solution, frozen immediately at -20°C or below); (≈) similar; ∆ altered, but not specified in which direction; (↑) increased; (↓) decreased; (h) hours; (d) days; (w) weeks; (y) years; RT, room temperature (approximately 25°C); accuracy (comparison of samples to samples without solution frozen immediately); stability (consistency of sample microbial composition over time). *Other abbreviations: iFOBT, immunochemical fecal occult blood test; PD, phylogenetic diversity*

**References**

1. Shaw AG, Sim K, Powell E, Cornwell E, Cramer T, McClure ZE, et al. Latitude in sample handling and storage for infant faecal microbiota studies: the elephant in the room? Microbiome. 2016;4(1):40.

2. Carroll IM, Ringel-Kulka T, Siddle JP, Klaenhammer TR, Ringel Y. Characterization of the fecal microbiota using high-throughput sequencing reveals a stable microbial community during storage. PLoS One. 2012;7(10):e46953.

3. Choo JM, Leong LE, Rogers GB. Sample storage conditions significantly influence faecal microbiome profiles. Scientific Reports. 2015;5: 16350.

4. Lauber CL, Zhou N, Gordon JI, Knight R, Fierer N. Effect of storage conditions on the assessment of bacterial community structure in soil and human-associated samples. FEMS Microbiol Lett. 2010;307(1):80-6.

5. Roesch LF, Casella G, Simell O, Krischer J, Wasserfall CH, Schatz D, et al. Influence of fecal sample storage on bacterial community diversity. Open Microbiol J. 2009;3:40-6.

6. Cardona S, Eck A, Cassellas M, Gallart M, Alastrue C, Dore J, et al. Storage conditions of intestinal microbiota matter in metagenomic analysis. BMC Microbiol. 2012;12:158.

7. Dominianni C, Wu J, Hayes RB, Ahn J. Comparison of methods for fecal microbiome biospecimen collection. BMC Microbiology. 2014;14(1):1.

8. Bassis CM, Moore NM, Lolans K, Seekatz AM, Weinstein RA, Young VB, et al. Comparison of stool versus rectal swab samples and storage conditions on bacterial community profiles. BMC Microbiol. 2017;17(1):78.

9. Stearns JC, Lynch MDJ, Senadheera DB, Tenenbaum HC, Goldberg MB, Cvitkovitch DG, et al. Bacterial biogeography of the human digestive tract. Scientific Reports. 2011;1:1-9.

10. Ott SJ, Musfeldt M, Timmis KN, Hampe J, Wenderoth DF, Schreiber S. In vitro alterations of intestinal bacterial microbiota in fecal samples during storage. Diagn Microbiol Infect Dis. 2004;50(4):237-45.

11. Guo Y, Li SH, Kuang YS, He JR, Lu JH, Luo BJ, et al. Effect of short-term room temperature storage on the microbial community in infant fecal samples. Sci Rep. 2016;6:26648.

12. Rounge TB MR, Nordby JI, Ambur OH, de Lange T, Hoff G. Evaluating gut microbiota profiles from archived fecal samples. BMC Gastroenterology. 2018;18:171.

13. Tedjo DI, Jonkers DM, Savelkoul PH, Masclee AA, van Best N, Pierik MJ, et al. The effect of sampling and storage on the fecal microbiota composition in healthy and diseased subjects. PLoS One. 2015;10(5):e0126685.

14. Wu GD, Lewis JD, Hoffmann C, Chen YY, Knight R, Bittinger K, et al. Sampling and pyrosequencing methods for characterizing bacterial communities in the human gut using 16S sequence tags. BMC Microbiol. 2010;10:206.

15. Loftfield E, Vogtmann E, Sampson JN, Moore SC, Nelson H, Knight R, et al. Comparison of collection methods for fecal samples for discovery metabolomics in epidemiological studies. Cancer Epidemiology Biomarkers & Prevention. 2016;25(11):1483-90.

16. Gudra D SS, Fridmanis D, et al. A widely used sampling device in colorectal cancer screening programmes allows for largescale microbiome studies. Gut. 2018;0:1–3.

17. Baxter NT, Koumpouras CC, Rogers MA, Ruffin MTt, Schloss PD. DNA from fecal immunochemical test can replace stool for detection of colonic lesions using a microbiota-based model. Microbiome. 2016;4(1):59.

18. Taylor MW. Examining the potential use and long-term stability of guaiac faecal occult blood test cards for microbial DNA 16S rRNA sequencing. J Clin Pathol. 2017;70:600-6.

19. Nechvatal JM, Ram JL, Basson MD, Namprachan P, Niec SR, Badsha KZ, et al. Fecal collection, ambient preservation, and DNA extraction for PCR amplification of bacterial and human markers from human feces. J Microbiol Methods. 2008;72(2):124-32.

20. Hale VL, Tan CL, Knight R, Amato KR. Effect of preservation method on spider monkey (Ateles geoffroyi) fecal microbiota over 8 weeks. J Microbiol Methods. 2015;113:16-26.

21. Wang Z ZC, Qiu Y, Usyk M, Wang T, Strickler HD, Isasi CR, Kaplan RC, Kurland IJ, Qi Q, Burk RD. Comparison of Fecal Collection Methods for Microbiome and Metabolomics Studies. Frontiers in Cellular and Infection Microbiology. 2018;8(301):1-10.

22. Franzosa EA, Morgan XC, Segata N, Waldron L, Reyes J, Earl AM, et al. Relating the metatranscriptome and metagenome of the human gut. Proc Natl Acad Sci U S A. 2014;111(22):E2329-38.

23. Vandeputte D, Tito RY, Vanleeuwen R, Falony G, Raes J. Practical considerations for large-scale gut microbiome studies. FEMS Microbiol Rev. 2017;41(Supp_1):S154-S67.

24. Kilpatrick CW. Noncryogenic preservation of mammalian tissues for DNA extraction: an assessment of storage methods. Biochem Genet. 2002;40(1-2):53-62.

25. Vlckova K, Mrazek J, Kopecny J, Petrzelkova KJ. Evaluation of different storage methods to characterize the fecal bacterial communities of captive western lowland gorillas (Gorilla gorilla gorilla). J Microbiol Methods. 2012;91(1):45-51.

26. Flores R, Shi J, Yu G, Ma B, Ravel J, Goedert JJ, et al. Collection media and delayed freezing effects on microbial composition of human stool. Microbiome. 2015;3:33.

27. Sinha R, Vogtmann E, Chen J, Amir A, Shi J, Sampson J, et al. Fecal Microbiome in Epidemiologic Studies—Response. Cancer Epidemiology Biomarkers & Prevention. 2016;25(5):870-1.

28. Chen Z, Hui PC, Hui M, Yeoh YK, Wong PY, Chan MCW, et al. Impact of Preservation Method and 16S rRNA Hypervariable Region on Gut Microbiota Profiling. mSystems. 2019;4(1):1-15.

29. Voigt AY, Costea PI, Kultima JR, Li SS, Zeller G, Sunagawa S, et al. Temporal and technical variability of human gut metagenomes. Genome Biol. 2015;16:73.

30. Hallmaier-Wacker LK, Lueert S, Roos C, Knauf S. The impact of storage buffer, DNA extraction method, and polymerase on microbial analysis. Sci Rep. 2018;8(1):6292.

31. Gorzelak MA, Gill SK, Tasnim N, Ahmadi-Vand Z, Jay M, Gibson DL. Methods for Improving Human Gut Microbiome Data by Reducing Variability through Sample Processing and Storage of Stool. PLoS One. 2015;10(8):e0134802.
